# Porphyrin-Polymer as a Photosensitizer Prodrug for Antimicrobial Photodynamic Therapy and Biomolecule Binding Ability

**DOI:** 10.1021/acs.biomac.4c01011

**Published:** 2024-11-08

**Authors:** Leila Tabrizi, Ross McGarry, Kaja Turzanska, Lazaros Varvarezos, Muireann Fallon, Ruairi Brannigan, John T. Costello, Deirdre Fitzgerald-Hughes, Mary T. Pryce

**Affiliations:** † School of Chemical Sciences, Dublin City University, Dublin D09W6Y4, Ireland; ‡ Clinical Microbiology, Royal College of Surgeons in Ireland, RCSI Education and Research, Beaumont Hospital, Beaumont, Dublin D09YD60, Ireland; § School of Physical Sciences, Dublin City University, Dublin 9 D09 K2WA, Ireland; ∥ Department of Physics, University of Ioannina, GR-45110 Ioannina, Greece

## Abstract

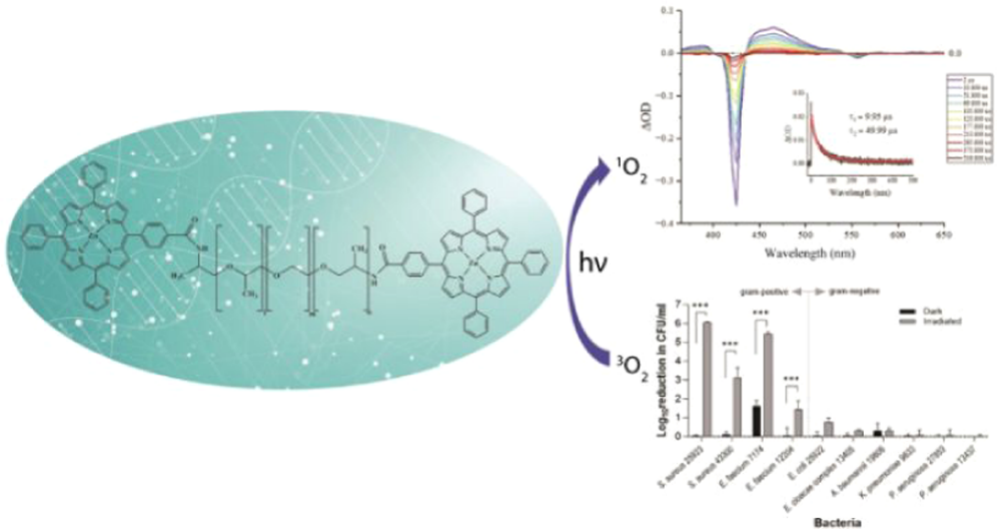

This study presents the development and characterization of a novel porphyrin-Jeffamine polymer conjugate designed to function as a photosensitizer prodrug for antimicrobial photodynamic therapy (aPDT). The conjugate features a photosensitive porphyrin unit covalently attached to a biocompatible polymer backbone, with enhanced solubility, stability, and bioavailability compared to those of the free porphyrin derivatives. The photophysical properties were studied using transient absorption spectroscopy spanning the fs–μs time scales in addition to emission studies. The production of reactive oxygen species upon photoactivation enabled effective bacterial cell killing. Spectroscopic studies confirmed strong binding of the conjugate to DNA through intercalation, likely disrupting DNA replication and transcription. Interaction studies with bovine serum albumin demonstrated substantial serum protein binding, which may positively impact the pharmacokinetics and biodistribution. Overall, this porphyrin-polymer conjugate offers a multifunctional theranostic platform, combining antimicrobial action with DNA and protein binding potential, positioning it as a promising candidate for aPDT and bioimaging applications.

## Introduction

The discovery and use of antibiotics has revolutionized modern medicine. However, the growing problem of antimicrobial resistance (AMR) threatens the gains made in treating infections and enabling life-enhancing medical interventions. Microorganisms, such as bacteria, can gain or acquire mechanisms of resistance against antimicrobials, rendering many groups of antibiotics ineffective in treating infections.^1^ A study by the World Health Organization (WHO) noted an increase in several AMR bacteria globally.^2^ It was estimated in 2019 alone that 4.95 million deaths were associated with infections caused by AMR bacteria.^3^ In addition, most clinically significant infections, particularly those associated with wounds and implanted medical devices, involve bacteria growing as biofilms, which are communities of bacteria encased in a protein/carbohydrate-rich adhesive matrix. Biofilm-associated infections are some of the most challenging ones due to the failure of most conventional antibiotics to penetrate the biofilm matrix and kill cells with a slower metabolism.

There is an urgent need for the development of novel antimicrobial and antibiofilm agents, especially those with alternative mechanisms of action to conventional antibiotics. Several authors have shown that antimicrobial photodynamic therapy (aPDT) effectively inactivates AMR bacteria,^4−6^ but its applicability in the management of AMR infections requires further exploration to realize its therapeutic potential.

Photodynamic therapy (PDT) is a photochemistry-based method that has been used clinically in cancer chemotherapy, with limited side effects compared to more traditional therapies.^7^ The three components required for PDT are a photosensitizer, a light source, and molecular oxygen.^8,9^ Porphyrins and their derivatives have attracted significant interest as photosensitizers in PDT because of their planar aromatic structure, photophysical properties, attractive absorption and emission properties, structural robustness, and high reactive oxygen species (ROS) yield upon irradiation.^10−12^ Compared to porphyrin compounds, porphyrin-based polymer analogues have notable benefits such as the excellent biocompatibility and structural stability, which further enhance the functionalities of porphyrins for a wide range of clinical applications.^13−17^

Zinc tetraphenylporphyrin (ZnTPP) was used in this study as opposed to free base tetraphenylporphyrin (TPP) for a number of reasons. Incorporation of zinc (d^10^) reduces aggregation tendencies and leads to a more stable photosensitizer. This is important as aggregation may reduce the quantum yield for singlet oxygen generation. Also, fluorescence may diminish when fourth-row metals are incorporated, thereby making ZnTPP ideal for applications requiring high singlet oxygen quantum yields and at the same time retaining fluorescence, if required for biological tracking studies.^18−20^

In recent years, researchers have focused on enhancing the efficacy and specificity of porphyrin-based photosensitizers for aPDT while minimizing the off-target effects and resistance development. One promising avenue is to design photosensitizing polymers as prodrugs, as this strategy not only enhances the pharmacokinetic profile of the photosensitizer but also opens up the possibility of targeted delivery to microbial cells. In addition to their antimicrobial activity, porphyrin–polymer conjugates exhibit interesting interactions with biomolecules such as DNA and proteins, which, with further exploration, can extend and augment their therapeutic and biomedical potential.^21−25^

Jeffamines are copolymers of ethylene oxide and propylene oxide in various ratios, including active amine groups with lower toxicity (monoamines, diamines, and triamines). They are commercially available with different Mw values and hydrophilic–lipophilic balance. Jeffamine-grafted copolymers with the ability to mediate cellular internalization and endosomal escape has been reported for delivery of specific gene silencing reagents.^26−29^

In this context, this study investigates a novel porphyrin-polymer in which Jeffamine, a linear polymer, is conjugated with ZnTPP, designed as a photosensitizing prodrug for aPDT. We have explored its antimicrobial activity against a range of pathogenic microorganisms and evaluated its DNA and bovine serum albumin (BSA) binding abilities. Through a comprehensive photophysical study, we investigated the excited-state dynamics, photostability, and singlet oxygen photogeneration efficiency of the conjugate, thereby providing valuable insights into its potential as a theranostic agent.

Overall, the integration of antimicrobial photodynamic therapy with DNA and protein binding capabilities in a single porphyrin-polymer conjugate presents a multifaceted approach to combating microbial infections.

## Experimental Section

### Materials and Instruments

All chemicals and solvents were supplied by Aldrich Chemicals Co. and anhydrous solvents containing sure/seal were used under the flow of nitrogen.

NMR spectra were recorded on a Bruker 600 MHz spectrometer. Fourier-transform infrared (FT-IR) measurements were carried out on a PerkinElmer 2000 FT-IR spectrophotometer in a liquid solution cell using spectrophotometric-grade chloroform. Gel permeation chromatography (GPC) was used to determine the dispersity (*Đ*_M_) and molecular weights of the synthesized polymers. GPC was conducted in THF using a PSS SecCurity2 system equipped with GPC precolumn PSS SDV analytical in THF, 50 mm × 8 mm, 5 μm columns in series, and a differential refractive index (RI) detector at a flow rate of 1.0 mL min^–1^. The systems were calibrated against Varian Polymer Laboratories Easi-Vial linear poly(styrene) (PS) standards and analyzed by the software package WinGPC 8.40. Mass spectrometry was conducted using a Waters LCT Premiere XE mass spectrometer. Electron spray ionization (ESI) (positive mode, Na+) and a time-of-flight (TOF) mass analyzer were employed. The samples were dissolved in acetonitrile (ACN).

### Ultrafast Spectroscopy

Ultrafast transient absorption spectroscopy (UTAS) measurements reported in this work were performed using a Transient Absorption Spectrometer, commercially available by Newport. The laser pulses were delivered by a Coherent Astrella Ti: Sapphire laser system. This arrangement provided high average power (7 W) pulses of ultrafast duration (∼40 fs measured by an intensity autocorrelator) at a repetition rate of 1 kHz and a wavelength centered at 800 nm. The second harmonic radiation, at 400 nm, generated by means of a Type I BBO crystal provided the pump beam. The incident (pump) laser pulse energy on the sample was 0.75 μJ, focused down to a spot of diameter of about 150–200 μm. The white light continuum (330–900 nm), generated by means of a CaF_2_ crystal, served as the probe beam. The polarization of the pump pulses at the sample was at a magic angle (54.7°) relative to the probe. The temporal resolution of the UTAS system was estimated to be ∼120 fs. To prevent photodegradation, measurements were performed using a combination of a rastering stage and flow-cell with a peristaltic pump (Fisherbrand CTP100). All samples were prepared and recorded in a demountable liquid cell (Harrick Scientific Products Inc., New York), with a 500 μm PTFE spacer and two 2-mm-thickness CaF_2_ windows (Crystran Ltd., Poole, UK). The optical density (OD) of each sample was ∼1 at the Soret band in a spectroscopy-grade solvent. Broadband transient absorption spectra were analyzed using version 1.5.1. of the Glotaran software package.^30−32^

### Nanosecond Transient Absorption Spectroscopy

Nanosecond transient absorption spectroscopy (ns-TAS) was recorded on an LP980 (Edinburgh Instruments) spectrometer. The excitation wavelength was set at 355 nm, and the pulse duration was approximately 2.5 ns. The samples were degassed using three freeze–pump thaw cycles and then placed under a nitrogen environment prior to the experiment. The incident laser pulse energy was measured as 8 mJ. The optical density of each sample was adjusted to ∼1 at the Soret band in the spectroscopic-grade solvent using a standard 1 cm path length quartz fluorescence cell. The optical density of each sample was recorded before and after the experiment to ensure that there was no photodegradation. Analysis of the results was performed using Edinburgh Instruments L900 software.

### Time-Correlated Single-Photon Counting

Time-correlated single-photon counting (TCSPC) measurements were recorded by using a FLS1000 (Edinburgh Instruments) spectrometer, where the excitation wavelength was set at 375 nm. Samples were degassed using three freeze–pump thaw cycles and then placed under a nitrogen environment prior to the experiment. The optical density of each sample was ∼0.2 at 375 nm, in a standard 1 cm path length quartz fluorescence cell. The UV–vis spectrum of each sample was compared before and after measurement to ensure no photodegradation. Analysis of the results was performed using Floracle software.

### Stability/Photostability of the Compounds

The stability of Jeff-ZnTPP was evaluated in 10% dimethyl sulfoxide (DMSO) (v/v in phosphate buffered saline (PBS)) at 37 °C. There was no detectable change in the UV–vis spectrum after 72 h, confirming the stability of the polymer under physiological conditions (Figure S17).

The photostability of the Jeff-ZnTPP polymer was evaluated by irradiation at 590 nm in THF. No detectable changes were observed in the UV–vis spectrum after 2 h of irradiation, confirming the polymer’s stability under these conditions (Figure S18). The experiment was repeated using irradiation at 420 and 660 nm, with the same results, further demonstrating the Jeff-ZnTPP polymer’s stability when irradiated.

### DNA Binding Studies

DNA binding studies were carried out with calf thymus (CT) DNA in 10 mM Tris-HCl/10 mM NaCl, buffer solution, pH = 7.2. Quality/purity of DNA stock solutions was assessed by *A*_260_/*A*_280_, which was ≥1.80. The bulk DNA solution was further diluted 1/10 and showed a maximum absorbance at 260 nm. The absorption coefficient of CT DNA was 6600 cm^–1^ M^–1^ per nucleotide.^33^ Solutions of compounds ZnTPP and Jeff-ZnTPP (10 μM) in Tris-HCl/NaCl were prepared. Aliquots of the complex solutions were transferred to cuvettes containing DNA over the range 0–24 μM, and changes in absorbance were measured.

Fluorescence spectroscopy, with ethidium bromide (EB) staining, was used to investigate the binding of ZnTPP or Jeff-ZnTPP to DNA. EB solution was prepared in a Tris-HCl/NaCl buffer (pH 7.2). Aliquots of 0–24 μM test solutions were added to DNA-EB (5 μM). The resulting solution was allowed to equilibrate for 2–5 min at room temperature. A change in the fluorescence intensity at 620 nm (450 nm excitation) was recorded.

### Protein Binding Studies

The binding of ZnTPP and Jeff-ZnTPP to BSA was investigated using fluorescence spectroscopy recorded at a fixed excitation wavelength corresponding to BSA at 280 nm and monitoring the emission at 335 nm at 298 K. The UV–vis absorption spectra were measured in the range of 200–800 nm at 298 K. The stock solution of BSA was prepared in 50 mM phosphate buffer (pH = 7.2) and stored in the dark at 4 °C for further use. Concentrated stock solutions of each test compound were prepared by dissolving the compound in phosphate buffer and then diluting with phosphate buffer to the required concentrations. Aliquots of 0–24 μM test solutions were added to the BSA (10 μM). A 2.5 mL solution containing BSA (10 μM) was titrated by successive additions of 0–24 μM test solutions (ZnTPP or Jeff-ZnTPP) for fluorescence or UV–vis measurements.

### Synthesis

#### Synthesis of Methyl Mono(*p*-carboxy)-tetraphenylporphyrin (**1**)

Synthesis of methyl mono(*p*-carboxy)-tetraphenylporphyrin (Me-pcTPP) (**1**) was prepared according to the literature procedure with slight modifications.^34^ Methyl 4-formylbenzoate (0.411 g, 2.5 mmol) was dissolved in dry DCM (100 mL), where benzaldehyde (0.795 mL, 7.5 mmol), pyrrole (0.695 mL, 10 mmol), and ground sodium chloride (0.0585 g, 1.00 mmol) were added. The solution was degassed with a stream of nitrogen under constant stirring at room temperature for 10 min. Boron trifluoride etherate (247 μL, 2.00 mmol) was added, and the reaction mixture was stirred for another 60 min until methyl 4-formylbenzoate was no longer evident by TLC analysis. 2,3-Dichloro-5,6-dicyano-1,4-benzoquinone (DDQ) (1.70 g, 7.5 mmol) was added, and the reaction was permitted to continue for an hour. Triethylamine (1.00 mL) was added to the reaction mixture, and the solvent was reduced in vacuo. The crude mixture was filtered over a short column of silica eluting with DCM followed by purification on silica eluting with DCM/hexane, 1:1. Me-pcTPP **1** was isolated from the second purple band on silica column and gave deep purple crystals in 19% yield (0.315 g, 0.467 mmol). ^1^H NMR (CDCl_3_): 8.76–8.84 (m, 8H), 8.40 (d, *J* = 7.9 Hz, 2H), 8.28 (d, *J* = 7.9 Hz, 2H), 8.17–8.19 (m, 6H), 7.69–7.75 (m, 9H), 4.11 (s, 3H), −2.79 (s, 2H). ^13^C NMR (CDCl_3_): 166.3, 146.0, 141.0, 140.9, 133.5, 128.5, 126.8, 125.6, 119.5, 117.4, 51.4.

#### Synthesis of Methyl Mono(*p*-carboxy)-tetraphenyl Zinc(II) Porphyrin Zn(II) (Zn-Me-pcTPP) (**2**)

Zn-Me-pcTPP (**2**) was prepared according to the literature procedure with slight modifications.^34^ Free base porphyrin **1** (200 mg, 0.31 mmol) and zinc acetate dihydrated (0.680 g, 3.10 mmol) were stirred overnight in 60 mL of CH_2_Cl_2_/MeOH (7:3) at 40 °C. The solution was then washed with water (3 × 20 mL), dried over MgSO_4_, and evaporated to dryness. The residue was taken up with a minimum amount of CH_2_Cl_2_ (5 mL) and precipitated by the addition of hexane (20 mL) to give **2** as a purple powder (205 mg, 100%). ^1^H NMR (CDCl_3_): *d* = 8.78–8.86 (m, 8H), 8.40 (d, *J* = 7.9 Hz, 2H), 8.25 (d, *J* = 7.9 Hz, 2H), 8.15–8.16 (m, 6H), 7.71–7.66 (m, 9H), 4.02 (s, 3H). ^13^C NMR (CDCl_3_): 167.4, 150.2, 149.4, 143.1, 134.5, 132.0, 131.8, 131.2, 129.1, 127.6, 126.4, 121.0, 52.3.

#### Synthesis of Zinc(II) 4-(10,15,20-Triphenylporphyrin-5-yl)benzoic Acid, Zn(II) (Zn-COOH-TPP) (**3**)

Lithium hydroxide monohydrate (3.11 mmol, 0.075 g) was added to a solution of methyl mono(*p*-carboxy)-tetraphenyl zinc(II) porphyrin Zn(II) (Zn-Me-pcTPP), **2** (220 mg, 0.311 mmol), in a THF/H_2_O (4:1) mixture (8 mL). The mixture was stirred at 0 °C for 12 h. The reaction mixture was divided between CH_2_Cl_2_ and 10% citric acid. The organic phase was washed with water, dried over MgSO_4_, and evaporated to dryness. Subsequently, the product was precipitated with CH_2_Cl_2_/hexane to quantitatively give **3** as a purple powder (230 mg, 100%). ^1^H NMR (*d*_6_-DMSO): *d* = 8.80–8.83 (m, 8H), 8.40 (d, *J* = 8.0 Hz, 2H), 8.34 (d, *J* = 8.0 Hz, 2H), 8.20–8.22 (m, 6H), 7.80–7.84 (m, 9H). ^13^C NMR (*d*_6_-DMSO): 168.0, 149.8, 149.3, 147.8, 143.1, 134.8, 134.6, 132.3, 131.8, 130.3, 127.9, 127.0, 121.1. TOF-MS: *m*/*z*: 743.1026 [M + Na]^+^.

#### Synthesis of Jeff-ZnTPP Polymer

Zn-COOH-TPP (0.253 mmol, 0.175 g) was suspended in dry dichloromethane (20 mL), while oxalyl chloride (2.53 mmol, 220 μL) was added, followed by two drops of DMF (as a catalyst). The mixture was stirred at 25 °C for 10 h. The solvent was removed under reduced pressure, and traces of oxalyl chloride were removed by the addition and subsequent evaporation of dichloromethane (2 × 3 mL). The residue was dissolved in dry THF (10 mL) and added to an ice cold solution of *O*,*O*′-bis(2-aminopropyl) polypropylene glycol-*block*-polyethylene glycol-*block*-polypropylene glycol1 (Jeffamine ED-900) (0.1265 mmol, 0.102 g) and triethylamine (1.265 mmol, 177 μL) in dry THF (10 mL). The mixture was stirred at 25 °C for another 8 h, concentrated under reduced pressure, and the residue was partitioned between CH_2_Cl_2_ (50 mL) and water (50 mL). The organic extract was washed with NaHCO_3_ 1% (2 × 50 mL) and water (2 × 50 mL) and dried over MgSO_4_. The solvent was removed under reduced pressure to give the final product (yield: 150 mg, 92%). ^1^H NMR (CDCl_3_): *d =* 9.05–9.20 (m, 8H), 8.34–8.52 (m, 9H), 7.98 (s, 10H), 0.25–3.38 (m, 126H). *M*_W_ = 2442 g/mol, *Đ*_M_ = 1.06 (RI detection, THF GPC).

#### Procedure for Singlet Oxygen Photogeneration

The 1.5 mL solution of porphyrin compounds or polymers (5 μM) in DMF was added into a 1.5 mL solution of 1,3-diphenylisobenzofuran (DPBF) (25 μM) in DMF in a quartz cell. The system was kept in a dark condition to avoid interference during irradiation. The solution was irradiated with simulated sunlight or 590 nm light source.^35^ Time-dependent electronic absorption spectra were recorded with a UV–vis spectrometer at certain time intervals. The absorption intensity of DPBF at 415 nm was used to estimate the ability for singlet oxygen generation. The formula used for the estimation of singlet oxygen quantum yield (Φ) was adapted from the following equation
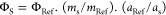
where the indexes s and ref indicate investigated Jeff-ZnTPP Polymer and ZnTPP, respectively, Φ is the quantum yield of singlet oxygen photogeneration; *m* is the slope of a trend line of change in the absorbance of DPBF (at 415 nm) vs time, and α is the absorption correction factor given by α = 1 – 10^–A^ (*A* is the absorbance at 590 nm).

#### Determination of Bactericidal Activity

##### Strains and Clinical Isolates Used

Initially, *Staphylococcus aureus* from the American Tissue Culture Collection ATCC25923 was used to determine the active concentration and other assay conditions. The activity spectrum was then determined using reference strains representing WHO-designated priority pathogens. These are pathogens with the acronym ESKAPE (*Enterococcus* spp., *S. aureus*, *Klebsiella pneumoniae*, *Acinetobacter baumannii*, *Pseudomonas aeruginosa*, and *Enterobacter* spp.). Reference strains were sourced from the ATCC or the National Collection of Type Cultures (NCTC), United Kingdom Health Security Agency (UK HSA). Where possible, an AMR strain and an antibiotic susceptible strain was included. Clinical isolates recovered from wound infections were also investigated and the collection was recently described.^36^ These were provided to the researchers anonymously following confirmation of their identity to species level by Matrix Assisted Laser Ionization Desorption Time-of-Flight Mass Spectrometry (MALDI-TOF-MS) using a Brüker Daltonik MALDI Biotyper. Ethical approval was obtained from Beaumont Hospital Ethics Committee (Reference number 22/17) for the use of these isolates. No patient information was collected. Further details of the strain and isolates used are provided in Table S2.

##### Photoactive Bactericidal Assay

Bacterial strains were grown overnight on Mueller–Hinton (MH) agar. Suspensions were prepared from isolated colonies to the density of a 0.5 McFarland standard (bioMèrieux, Ireland) and were further diluted 1/100 in sterile PBS, to a final concentration of approximately 1 × 10^5^ colony forming units (CFU)/mL. Stock concentrations of 10 mg/mL ZnTPP and Jeff-ZnTPP polymer were prepared in sterile 100% DMSO (v/v in PBS) and stored in glass containers protected from light at 5 °C for up to 1 week. Under sterile conditions, 180 μL of bacterial suspension (1 × 10^5^ CFU/mL) was added to wells of two 96-well plates (Nunc, Denmark). ZnTPP and Jeff-ZnTPP polymer stocks were further diluted to 1 mg/mL, 10% DMSO and vortex-mixed until dissolved. ZnTPP or polymer (20 μL) was added to wells in duplicate for a final assay volume of 200 μL and a final assay concentration of 100 μg/mL. Untreated controls contained 10% DMSO instead of the test agents. One 96-well plate was exposed to an LED light source suspended 25 cm above the plate for the required irradiation time at room temperature. The light source was a dichromatic lamp, λ = 420 and 660 nm, 21.93 mW/cm^2^. A radiometer (Delta Ohm HD 2101.2) equipped with an irradiance probe (LP 471 RAD) was used to measure the fluence rate of the dichromatic lamp. The second 96-well plate was incubated in the dark at room temperature. After incubation, samples were pipet-mixed to ensure homogeneity. Bacterial viability was determined using the Miles and Misra method.^37^ Briefly, serial dilutions of samples were made in sterile PBS. Three 20 μL aliquots of each dilution were spot-plated on MH agar and incubated statically overnight at 37 °C. Following incubation, colony forming units (CFU)/mL was calculated, and the log_10_ reduction in CFU/mL, compared to untreated controls, was determined for each treatment. The temperature was measured during irradiation and ranged from 30 to 33 °C.

##### Biocompatibility Studies

Potential cytotoxicity to human cells was investigated by determining the metabolic activity in keratinocytes in culture and hemolytic activity in primary human erythrocytes. As described previously,^38^ human HaCaT cells (aneuploid immortal keratinocyte cell line) were cultured in Dulbecco’s modified Eagle medium (Gibco) supplemented with 10% fetal bovine serum. Cells were seeded at 3 × 10^5^ cells/mL for 24 h at 37 °C before incubation with doubling dilutions of Jeff-ZnTPP from 800 to 1.56 μg/mL in a final volume of 10% DMSO (v/v) for 60 min in dark conditions only (light conditions were not investigated in this case as this would require removal of the plates from the CO_2_ incubator for prolonged periods). Triton-X-100 (2%, v/v) was used as a positive control, and culture media alone was used as a negative control. Media were removed, and the cells were washed and incubated with 100 μL of 500 mg/L MTT (3-[4,5-dimethyl-2-thiazolyl]-2,5-diphenyl-2*H*-tetrazolium bromide; Sigma) for 4 h, protected from light. DMSO was added to solubilize, and plates were covered with aluminum foil and agitated on an orbital shaker for 5–10 min. The absorbance was read at 590/595 nm using a plate reader (VICTOR X3 2030 Multilabel Reader, PerkinElmer). For estimation of hemolysis, the method of Zapotoczna et al. was used.^38^ Briefly, healthy human volunteer blood was drawn into tubes containing EDTA; (1.6 mg/mL). Erythrocytes were separated by centrifugation at 1000*g* for 5 min at 18 °C and were washed twice with PBS. The washed pellet was resuspended to five times the original volume of blood collected. In a 96-well plate, 50 μL of the erythrocyte suspension was mixed with 50 μL of ZnTPP or Jeff-ZnTPP in 10% DMSO. Positive control (100% hemolysis) contained Triton-X-100 (2%, v/v), and negative controls (0% hemolysis) contained PBS instead of porphyrins. After 60 min of incubation at 37 °C, the plates were centrifuged for 5 min at 500*g*. The supernatant was removed into new 96-well plates and the absorbance was measured with a plate reader (VICTOR X3 2030 Multilabel Reader, PerkinElmer) at a wavelength of 405 nm. For both biocompatibility assays, retention of metabolic activity (MTT assay) and % hemolysis was estimated with reference to controls. Ethical approval with explicit consent for human volunteer blood was obtained from the Royal College of Surgeons Research Ethics Committee, reference number 202203014.

## Results and Discussion

### Synthesis and Characterization

**Scheme 1:**
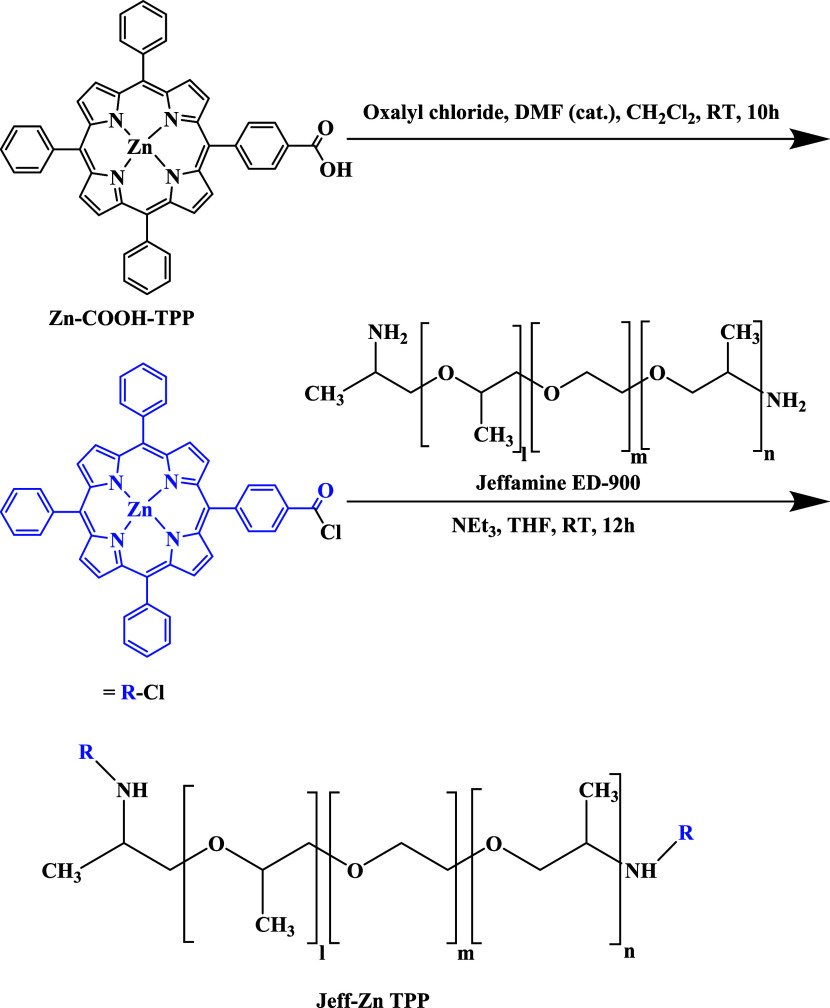
Synthetic Route to Jeff-ZnTPP Polymer

For the synthesis of the porphyrin building block **3**, Me-pcTPP, **1**, was used as the starting porphyrin (Scheme S1). **1** was easily accessible using the Lindsey’s method of a one-pot two-step reaction of pyrrole, benzaldehyde, and methyl 4-formylbenzoate under the BF_3_·OEt_2_ catalysis followed by DDQ oxidation. The reaction was modified by using 0.1 M NaCl solution in DCM as this approach improved the isolated yield of **1**.^39^ For the synthesis of Zn(II)-porphyrin (Scheme 1), Zn-Me-pcTPP, (**2**), zinc was then inserted by treating **1** with Zn(OAc)_2_ in CH_2_Cl_2_/MeOH (7:3) under reflux.^34^ Hydrolysis of its methyl ester group was achieved by treatment with LiOH in a THF/H_2_O (4:1) mixture. After an acidic workup with citric acid, the desired Zn-COOH-TPP (**3**) was obtained (Scheme S1).

The Jeff-ZnTPP polymer was prepared by the condensation reaction of Zn-COOH-TPP, **3**, with oxalyl chloride in the first step, followed by coupling Jeffamine ED-900 (Sigma-Aldrich) in the second step in dry DCM and using TEA as a proton acceptor. The reaction pathways are listed in Scheme 1.

The synthesis of Me-pcTPP (**1**) was confirmed by ^1^H NMR and UV/vis spectroscopy (Figures S1 and S13).^34^ In the next step, **1** was metalated using zinc acetate dihydrate to yield Zn-Me-pcTPP (**2**). UV/vis spectroscopy confirmed the successful conversion of Me-pcTPP into Zn-Me-pcTPP (**2**), as shown by the change in the Q-band absorption profile (Figure S14), while the final Zn-COOH-TPP (**3**) was confirmed by the absence of the methyl hydrogen signal of methyl mono(*p*-carboxy) tetraphenylporphyrin at 4.02 ppm in the ^1^H NMR spectrum (Figure S5). The ESI-MS spectrum of Zn-COOH-TPP shows peaks centered at *m*/*z* 743.1026 ((M + Na) +) with the agreement of simulated patterns and structures (Figure S7).

The synthesis of the Jeff-ZnTPP polymer was confirmed using ^1^H NMR spectroscopy, with the porphyrin peaks in Jeff-ZnTPP occurring in the range of 7.97–9.20 ppm (Figure S9). In the Jeff-ZnTPP polymer, the peaks at 0.24–3.38 ppm were attributed to the −CH_2_– and −CH_3_ protons within the Jeffamine backbone. In the FT-IR spectrum, a new vibration at ∼1600 cm^–1^ was assigned to the C=O stretching mode of the amide in the Jeff-ZnTPP polymer.

Gel permeation chromatography (GPC) results of Jeff-ZnTPP polymer reveal that the average weight molar mass (*M*_w_) is 2442, while the average number molecular mass (*M*_n_) is 2310 g/mol. The yielding polydispersity value (*Đ*) for the Jeff-ZnTPP polymer is thus 1.06. The *M*_w_ and *M*_n_ for Jeffamine were 1154 and 1061 g/mol, respectively (Figure S16 and Table S1).

FT-IR spectra of Jeffamine and Jeff-ZnTPP polymers in chloroform were recorded (Figure S12). In the FT-IR spectrum of the Jeffamine polymer, a characteristic peak for the NH_2_ group appears in the range of 3250–3400 cm^–1^, indicating the presence of primary amine groups at the polymer ends. This peak was not present in the spectrum of the Jeff-ZnTPP polymer, confirming that the reaction occurred at both polymer chain ends. GPC also supports the conclusion. The Jeffamine polymer showed a molecular weight peak (*M*_w_) of 1154 g/mol, which is consistent with the unreacted polymer. After the ZnTPP reaction, this peak disappeared, and a new peak at Mw 2442 g/mol appeared in the Jeff-ZnTPP polymer, corresponding to the successful modification with ZnTPP. The fact that the GPC profile shows a single, sharp peak suggests the formation of a well-defined product, indicating that both ends of the polymer underwent the reaction with no significant unreacted material remaining. The disappearance of the NH_2_ peak in FT-IR and the shift in molecular weight in GPC confirm that both ends of the polymer successfully underwent the ZnTPP reaction without additional purification steps.

### Singlet Oxygen (^1^O_2_) Production Measurements

The capacity of the porphyrin compounds to generate singlet oxygen under irradiation is a condition for photodynamic therapy, and the singlet oxygen yield verifies the potential capacity of photosensitizers. 1,3-Diphenylbenzofuran (DPBF) is an excellent singlet oxygen scavenger.^40^ The ability of ZnTPP and Jeff-ZnTPP polymer to generate singlet oxygen was detected by time-dependent electronic absorption spectroscopy to control the change of absorbance of DFBF at 415 nm during different irradiation times using a 590 nm LED source. Displayed in Figure S19 are the changes observed in the UV–vis spectra with the generation of singlet oxygen evident by the decreasing absorption band of DPBF at 415 nm upon. Figure S17 (inset) is obtained by plotting *A*/*A*_0_ versus time, where A is the absorbance at 415 nm for a certain time in the presence of porphyrins or polymers, and *A*_0_ is the initial absorbance. The quantum yields of singlet oxygen generation (Φ_Δ_) were 0.70 and 0.75 for Jeff-ZnTPP and ZnTPP (zinc tetraphenyl porphyrin, as standard for comparison^35^) in DMF, respectively.

### Time-Resolved Photophysical Measurements

**Figure 1:**
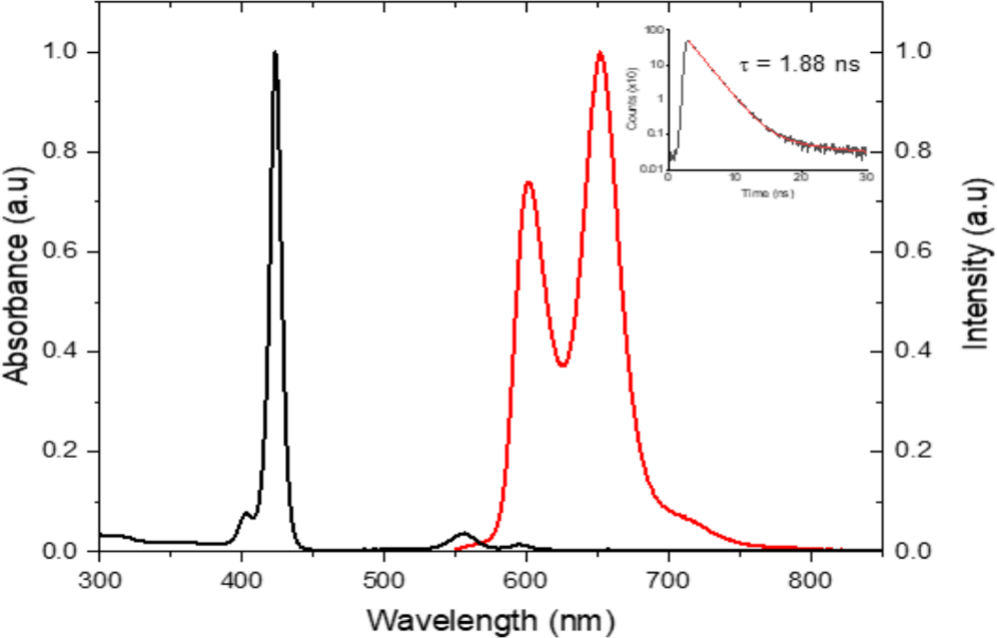
Normalized UV–vis absorption and emission spectra of Jeff-ZnTPP in THF. The inset displays the fluorescence decay profile of Jeff-ZnTPP in THF over a 30 ns time range. The red solid curve represents the fit to the measured data points.

The UV–vis and emission spectra of Jeff-ZnTPP in THF are presented in Figure 1. The compound exhibits a broad absorbance in the visible region (400–620 nm) mapping that of ZnTPP. The strong absorbances noted at 403/424 nm (Soret Band) and 555/595 nm (Q-Band) correspond to *S*_0_ – *S*_3_/*S*_4_ and *S*_0_ – *S*_1_/*S*_2_ transitions, respectively. The compound is fluorescent (λ_max_ = 655 nm), arising from *S*_1_ – *S*_0_ relaxation. To further understand the underlying photophysical processes, time-resolved measurements were performed over a broad range of time scales.

**Figure 2:**
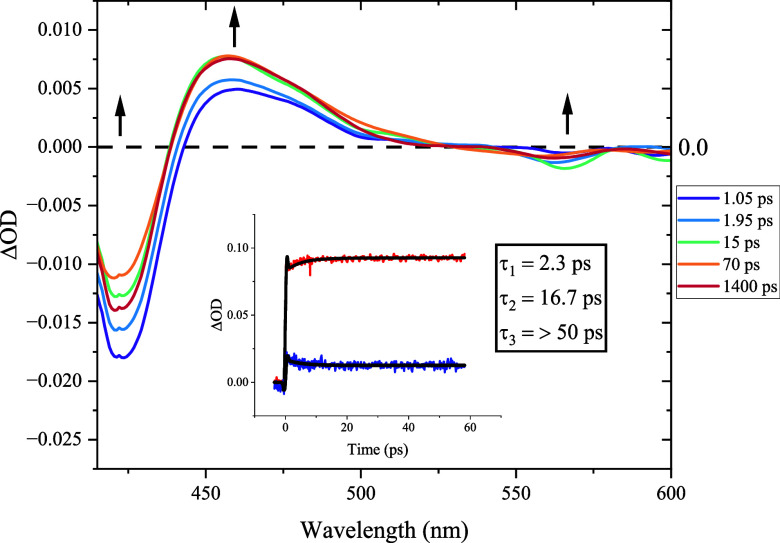
Spectral transient absorption profile for Jeff-ZnTPP in THF, following excitation at 400 nm. The inset displays the kinetic time traces for Jeff-ZnTPP at 450 nm (red data points) and 550 nm (blue data points) for a time delay range of 0 to >50 ps.

Ultrafast transient absorption measurements were performed in two solvents, namely, tetrahydrofuran (THF) (Figure 2) and ethyl acetate (EA) (Figures S20–S22). Figure 2 displays the resulting ultrafast transient absorption spectra for Jeff-ZnTPP. Regions of interest are indicated with arrows. The kinetic decay profiles for two wavelengths (450 and 650 nm) are also displayed for Jeff-ZnTPP. The red data points represent a decay profile recorded at 450 nm, and blue data points correspond to a decay curve recorded at 650 nm. The solid black line represents the fitted curve following global analysis of the obtained spectra.

Jeff-ZnTPP exhibits a similar spectral profile on the ultrafast time scale, to the spectra obtained for the extensively studied zinc tetraphenyl porphyrin.^41^ The changes in absorbance spectra following excitation are displayed in Figure 2, with key changes denoted by black arrows. The bleaches at 424, 560, and 590 nm correspond to the depletion of the Soret and Q-bands, respectively, and are fully realized after 700 fs. Further spectral changes are observed at ca. 450–550 nm, with a slight increase in intensity and narrowing of the excited-state absorption (ESA) band up to 30 ps. Following this, there is little change observed in the ESA features over the remainder of the experimental window (1.5 ns), with only a marginal recovery in the ground-state bleach being noted. For Jeff-ZnTPP, three time constants were obtained: τ_1_ = 2.3 ps, τ_2_ = 16.7 ps, and τ_3_ > 1500 ps (in THF). These lifetimes are in agreement with the previously reported work by Zewail and co-workers on ZnTPP.^43^ Upon excitation at 400 nm, the *S*_2_ state is rapidly populated and subsequently undergoes internal conversion (τ_1_) to the vibrationally hot *S*_1_ state. The *S*_1_ state in turn undergoes vibrational cooling (τ_2_) and persists (τ_3_) for the remainder of the experimental window. The lifetimes for this latter component were measured using a combination of TCSPC and nanosecond-TAS experiments (see Figure 4).^42^

Time-correlated single-photon counting was used to measure the fluorescence lifetime *S*_1_ → *S*_0_ for Jeff-ZnTPP in both ethyl acetate and THF. Deconvolution was carried out by iterative reconvolution of the instrument response function (IRF). The accuracy of the fit was judged by the chi-squared value (χ^2^, between 1.1 and 1.3) and by the sum of the residuals. A monoexponential decay was observed in both solvents, resulting in fluorescence lifetimes of 1.88 ns (THF) and 2.25 ns (ethyl acetate).^43^ There is a slight increase in the fluorescence lifetime of the porphyrin-polymer in the more polar ethyl acetate, compared to THF. This is due to the solute–solvent stabilization effect on the S_1_ excited state.^44^ The more polar solvent can better stabilize the moderate dipole moment of the *S*_1_ excited state, allowing it to remain in the excited state for longer, resulting in an increased fluorescence lifetime.

**Figure 3:**
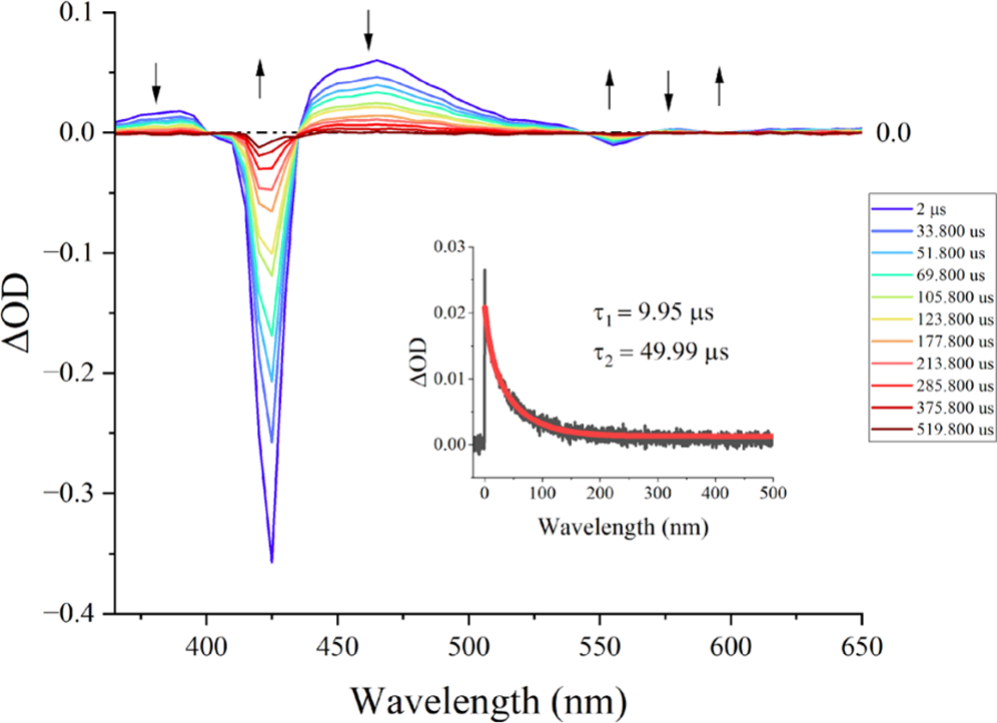
Spectral transient absorption profile for Jeff-ZnTPP in ethyl acetate over a 500 μs time delay following excitation at 355 nm. The inset displays the kinetic time trace for Jeff-ZnTPP at 550 nm.

Nanosecond-TAS measurements were performed to investigate the polymeric porphyrin for the presence of a triplet lifetime. Two solvents, ethyl acetate and tetrahydrofuran were used, where the experiments were carried out under both a nitrogen and air atmosphere, following excitation at 355 nm. Figure 3 displays a set of representative spectral transient absorption profiles for Jeff-ZnTPP with regions of interest denoted with arrows. The kinetic traces recorded at 550 nm are also displayed for Jeff-ZnTPP, with the fitted line displayed (red curve).

**Figure 4:**
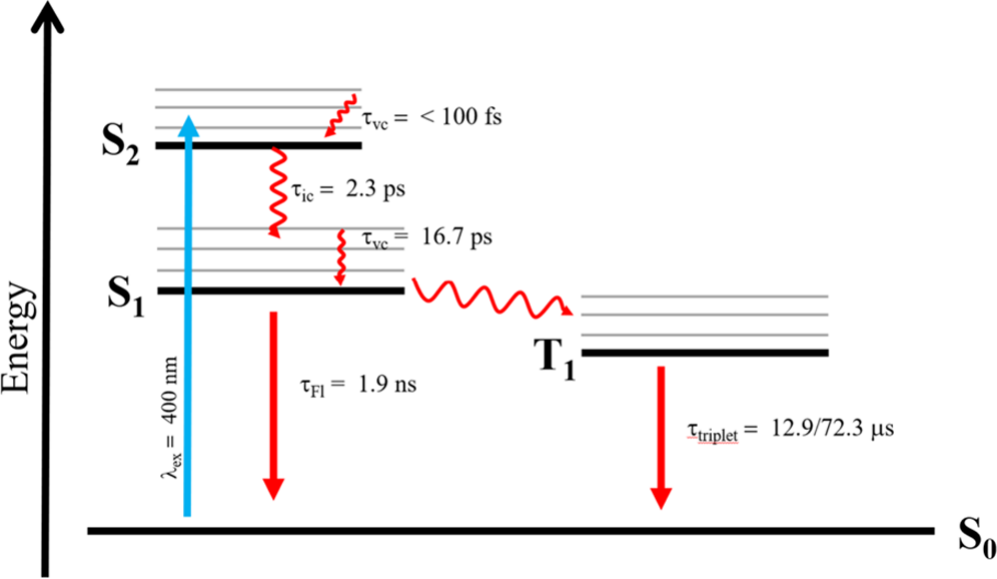
Jablonksi diagram summarizing the observed photophysical processes following excitation of Jeff-ZnTPP in THF.

The nanosecond TA spectrum of Jeff-ZnTPP displays GSBs (ground-state bleaches) at 425, 550, and 590 nm, corresponding to bleaching of the Soret band and the two Q-bands, respectively, in the porphyrin. ESA signals at 460 and 570 nm were also observed. The decay curves recorded at 550 nm, in ethyl acetate, were determined to have two lifetimes, τ_1_ = 9.95 μs and τ_2_ = 49.66 μs. These values increase to τ_1_ = 12.94 μs and τ_2_ = 72.26 in THF. The component τ_1_ represents 27% of the signal contribution in ethyl acetate and increases in THF to a 30% contribution. When compared to the reference compound ZnTPP, it is notable that Jeff-ZnTPP features a significantly increased lifetime (30 μs vs 72 μs in THF). This increase is attributed to the influence of the polymer, specifically, the influence of folding on the porphyrin itself. When photosensitizers are encapsulated in large nonconjugate systems, such as dendrimers,^45^ noticeable increases in lifetimes are reported, compared to the free photosensitizer, as these large systems shield the photosensitizer from various quenching processes.^46^ Furthermore, the nature of the polymer leads to microheterogeneous environments, which may result in different lifetimes observed by us and others. Therefore, the two lifetimes reported for this system are attributed to a mixture of different local environments in which the porphyrin exists, some being more shielded, thus enhancing the triplet lifetime. An overview of the various photophysical processes and the associated lifetimes are displayed in Figure 4.

### DNA Binding Studies

#### UV–vis Absorption Spectroscopy

**Figure 5:**
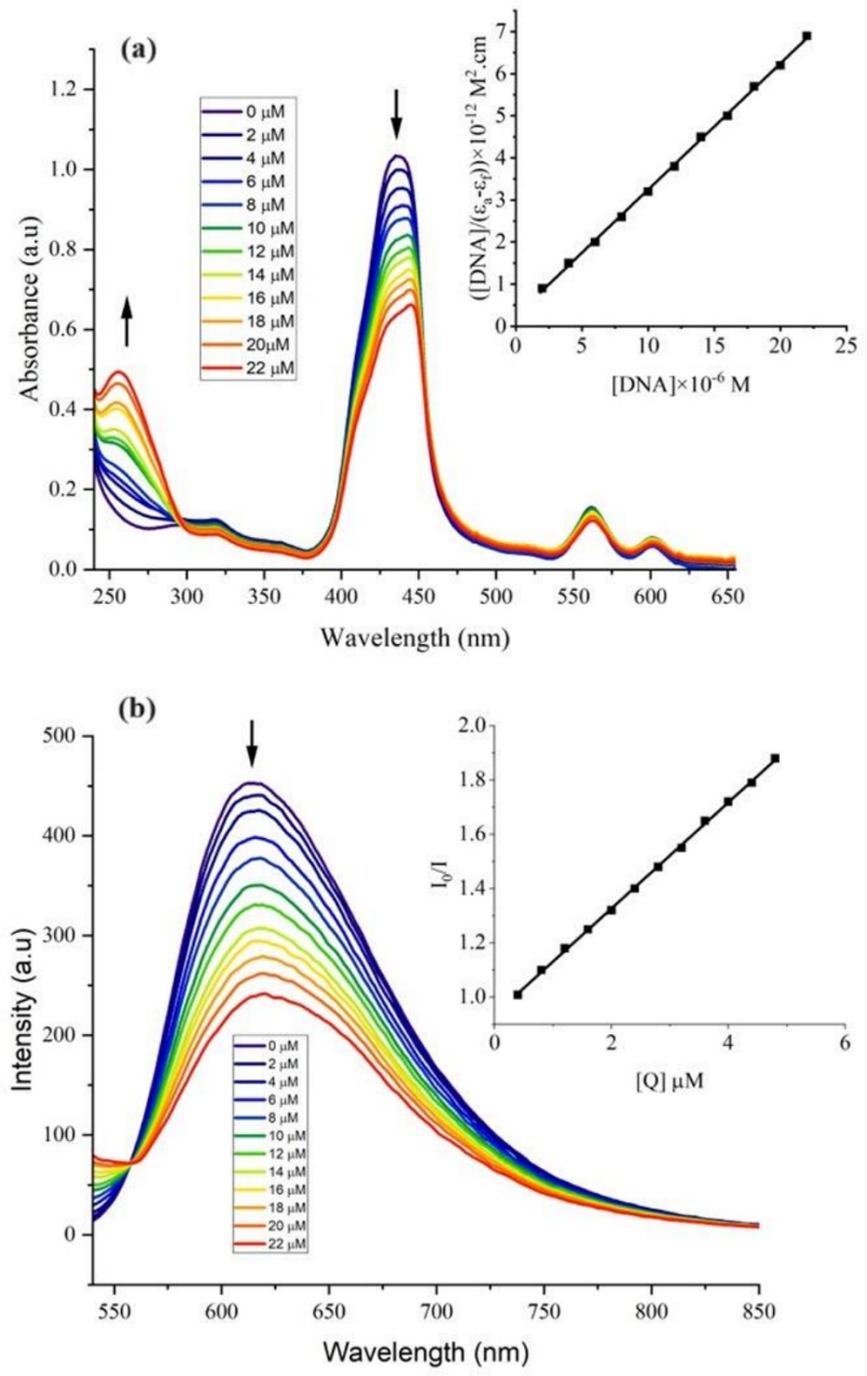
(a) Absorption spectra of Jeff-ZnTPP polymer (10 μM) and in Tris-HCl buffer upon addition of CT DNA (0–24 μM). Inset: Plot of [DNA]/(ε_a_ – ε_f_) versus [DNA] for the titration of the Jeff-ZnTPP polymer and with CT DNA. (b) Fluorescence quenching curves of EB bound to DNA in the presence of Jeff-ZnTPP in Tris-HCl buffer. [DNA] = 5 μM, [EB] = 5 μM, and [compounds] = 0–24 μM. Inset: Stern–Volmer plot of fluorescence titrations of Jeff-ZnTPP polymer with CT DNA.

Damage to the bacterial DNA of *S. aureus* and *E. coli* occurs during the photodynamic process as shown by Nir et al.^47^ In another study,^48^ alterations to the electrophoretic patterns of *S. aureus* chromosomal/plasmid DNA occurred following photodynamic killing with hematoporphyrin but the DNA damage occurred as a secondary effect post-killing, and the photodynamic process for other bacteria also confirmed this.^49^ We sought to determine the direct interaction of ZnTPP and Jeff-ZnTPP with calf thymus (CT) DNA as a model. The binding of metal complexes or organic compounds to CT DNA is frequently studied using UV–vis spectroscopy. This approach allows researchers to monitor changes in the absorption spectra, which can indicate interactions between the compounds and the DNA. With increasing addition of CT DNA (0–25 μM), the Soret band of ZnTPP and Jeff-ZnTPP displays hypochromism (a decrease in the absorbance of the Soret band), accompanied by a marginal red shift of 5 nm for Jeff-ZnTPP (Figures 5a and S23).

While noncationic porphyrins lack the potential for cation–anion electrostatic binding to DNA phosphate groups or amino acid residues, these porphyrins exhibit an overall shift in the Soret band absorption, corroborating with previous studies.^50^ It is well-known that complex binding to DNA through intercalation usually results in hypochromism with a red shift (typically in the range of 5–10 nm) of the Soret band of the UV–vis spectrum.^51^ Therefore, DNA binding with ZnTPP or the Jeff-ZnTPP polymer in our studies is attributed to the intercalative mode involving a strong stacking interaction between the aromatic chromophore and the base pairs of DNA.

The binding constant of the complexes with CT DNA (*K*_b_) was obtained from the ratio of the slope to intercept in plots [DNA]/(ε_a_ – ε_f_) versus [DNA] according to the equation^52^
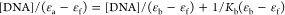
where ε_a_, ε_f_, and ε_b_ correspond to *A*_obsd_/[M], the extinction coefficient for free compounds, and the extinction coefficient for the compounds in the fully bound form, respectively. A plot of [DNA]/(ε_a_ – ε_f_) versus [DNA] gives a slope of 1/(ε_b_ – ε_f_) and a *y*-intercept equal to 1/*K*_b_ (ε_b_ – ε_f_); *K*_b_ is the ratio of the slope to the *y*-intercept (inset: Figures 5a and S23).

The binding constants obtained for ZnTPP and Jeff-ZnTPP polymer are (3.54 ± 0.02) × 10^4^ M^–1^ and (2.85 ± 0.02) × 10^5^ M^–1^, respectively. These *K*_b_ values indicate that the Jeff-ZnTPP polymer has a stronger binding affinity for CT DNA compared to that of ZnTPP.

#### Ethidium Bromide-DNA Fluorescence Quenching

To provide further evidence for the intercalation mode, the binding of ZnTPP and the Jeff-ZnTPP polymer to CT DNA were studied by a competitive binding fluorescence assay using ethidium bromide (EB). In our experiments, addition of various amounts of ZnTPP and Jeff-ZnTPP polymer to DNA pretreated with EB caused an appreciable reduction in the fluorescence emission intensity (Figures 5b and S24) due to the replacement of the EB fluorophore in the EB-DNA adduct by the complexes with an appropriate decrease in the emission intensity.^53^

With increasing concentrations of ZnTPP or Jeff-ZnTPP polymer, the intensity of the emission band at 620 nm of the EB-DNA system decreases. The observed linearity in the plot of *I*_o_/*I* vs the concentration ratio of the complexes to DNA is in good agreement with the linear Stern–Volmer equation^54^
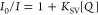
1where *I*_0_ and *I* are the fluorescence intensities exhibited in the absence and presence of the compounds, respectively; [*Q*] corresponds to the concentration ratio of the compound to DNA. The slope of the plot of *I*_0_/*I* versus [*Q*] gives *K*_SV_ (inset: Figures 5b and S24). The Stern–Volmer constant, *K*_SV_, was calculated to be (4.16 ± 0.02) × 10^4^ M^–1^ for ZnTPP and (1.95 ± 0.02) × 10^5^ M^–1^ for Jeff-ZnTPP. The quenching constant (*K*_q_) can be obtained from the equation *K*_SV_ = *K*_q_τ_0_, where τ_0_ is the lifetime of the fluorophore (CT-DNA-EB) in the absence of the quencher (19.2 ns).^55^*K*_q_ was calculated to be (2.17 ± 0.02) × 10^12^ M^–1^ s^–1^ for ZnTPP and (10.15 ± 0.02) × 10^12^ M^–1^ s^–1^ for Jeff-ZnTPP. Values of *K*_q_ in the range of 10^12^–10^13^ M^–1^ s^–1^ indicate static quenching due to the formation of the nonfluorescent adducts of the (EB-DNA)-porphyrin.^56^ The quenching constant for Jeff-ZnTPP is approximately a 5-fold increase compared to that of ZnTPP and agrees with the observation in the UV–vis studies discussed above.

The values obtained for *K*_q_ and *K*_SV_ for ZnTPP and Jeff-ZnTPP are consistent with the UV–vis titration results, suggesting that Jeff-ZnTPP intercalated strongly to DNA in comparison with ZnTPP. Although EB is a well-established intercalative binder, the displacing molecule is not necessarily an intercalator itself, since the only requirement is that it binds to DNA more firmly than EB in the same site or around it (between two adenine–thymine (A–T) pairs). Thus, this assay does not provide conclusive information about the binding mode but it does confirm the strength of the interaction.

Several factors may be responsible for the enhanced binding capability of the Jeff-ZnTPP polymer compared with ZnTPP, including the increased surface area of Jeff-ZnTPP or the flexible conformation in the Jeff-ZnTPP polymer. Further studies are required to confirm our observations. Direct DNA binding effects of Jeff-Zn or ZnTPP are likely to play only a secondary role in its antimicrobial activity. Direct DNA damaging effects also assume cellular uptake of the molecule into the cell, which is highly species-dependent. Nonetheless, the enhanced binding in the porphyrin-polymer conjugate highlights the potential for such assemblies as promising candidates for other biomedical applications such as DNA sensing, drug delivery, and photodynamic anticancer therapy.^57−61^

#### Protein Binding Studies

Serum albumin is the most abundant protein in blood, and among its roles are the carriage of biomolecules to improve their stability and reduce their toxic effects while in circulation. The interaction of potential therapeutics with this protein is important in drug development and prediction of their pharmacokinetic properties. Since the interactions of antibiotics and other drugs with BSA were shown to be similar to those with human serum albumin (HSA),^62,63^ BSA was used here as a model protein for Jeff-ZnTPP–protein interaction studies. The interaction of ZnTPP and Jeff-ZnTPP with BSA was investigated by fluorescence spectroscopy, which is an effective method to qualitatively analyze the binding of complexes to BSA. Generally, the native fluorescence of BSA is attributed to tryptophan, tyrosine, and phenylalanine residues. The fluorescence of BSA at 348 nm was mainly attributable to the amino acid tryptophan in the macromolecule.^64−66^

**Figure 6:**
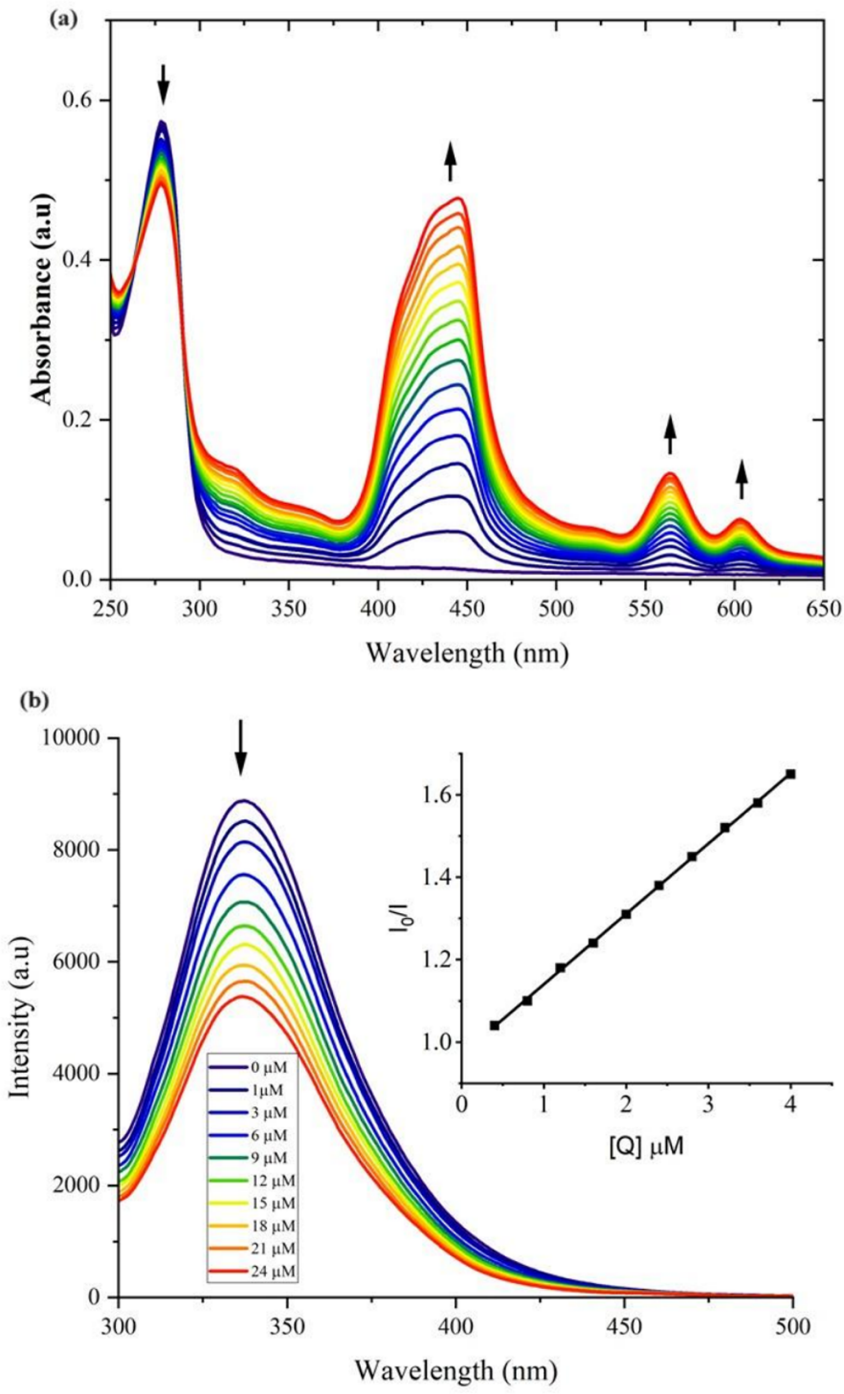
(a) UV–vis absorption of BSA (10 μM) in PBS solution in the presence of different amounts (0–24 μM) of Jeff-ZnTPP polymer. (b) Fluorescence quenching curves of BSA (10 μM) in PBS solution in the presence of different amounts (0–24 μM) of Jeff-ZnTPP polymer. Inset: Stern–Volmer plot of fluorescence titrations.

UV–vis absorption studies are a simple method to explore the structural changes of BSA.^67^ If static quenching occurs, the maximum absorption shifts due to conformation changes in the ground state of the complex, while no shift is assigned to the dynamic quenching process.^68^ From the absorption spectra (Figures 6a and S25), upon the addition of Jeff-ZnTPP or ZnTPP into the solution containing BSA, the UV–vis BSA absorption band around 278 nm decreases, with a significant increase in both the Soret and Q-bands centered at 440 nm and between 500 and 700 nm, respectively. Furthermore, the maximum absorption peak of BSA (278 nm) shifted toward a slightly higher wavelength (3 nm), which means the ground-state complex was formed between BSA and ZnTPP or Jeff-ZnTPP.^69^

Displayed in Figures 6b and S25 are the fluorescence emission spectra of BSA in the presence of different concentrations of Jeff-Zn and ZnTPP, respectively. The emission intensity of BSA decreased with the addition of Jeff-ZnTPP or ZnTPP, and this observation suggested that both compounds were interacting with BSA at the proximity of tryptophan residues. The Stern–Volmer constant, K_SV_, was calculated to be (7.52 ± 0.02) × 10^4^ M^–1^ for ZnTPP and (1.70 ± 0.02) × 10^5^ M^–1^ for Jeff-ZnTPP (inset Figures 8 and S26). The quenching constant (*K*_q_) where τ_0_ is the lifetime of the fluorophore (BSA) in the absence of the quencher (10 ns)^70^ yielded *K*_q_ = 7.52 ± 0.02 × 10^12^ M^–1^ s^–1^ for ZnTPP and 17 ± 0.02 × 10^12^ M^–1^ s^–1^ for Jeff-ZnTPP. The *K*_q_ value for Jeff-ZnTPP was double that obtained for the ZnTPP static model quenching process.^51^

For a static quenching interaction, the fluorescence intensity data can also be used to determine the apparent binding constant (*K*_b_) and the number of BSA binding sites (*n*) for the complex using the following equation^71,72^
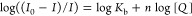
2

From the plot of log ((*I*_0_*– I*)/*I*) vs log [*Q*] (Figure S27), the number of binding sites (*n*) and binding constant (*K*_b_) values were calculated.

For both compounds, a linear relationship is observed with binding constants *K*_b_ of 1.1 × 10^6^ and 5.1 × 10^4^ M^–1^, respectively, for Jeff-ZnTPP and ZnTPP. In both compounds, the values of *n* were determined to be 1.08 and 1.04 for Jeff-ZnTPP and ZnTPP, respectively. As both values are close to 1, this indicates only one binding site for both Jeff-ZnTPP and ZnTPP on the BSA molecule.

The results demonstrate that Jeff-ZnTPP interacts strongly with BSA in comparison with ZnTPP. The increase in the surface area of Jeff-ZnTPP or the flexible conformation in the Jeff-ZnTPP polymer may contribute toward this enhancement.^73−76^

#### Antimicrobial Activity Investigation

**Figure 7:**
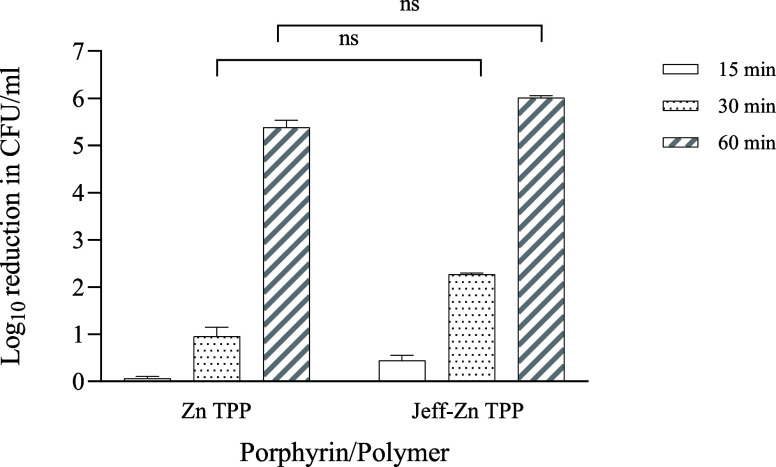
Time dependence of light-activated bacterial killing for Jeff-ZnTPP polymer vs ZnTPP. Data shown are the log reduction in CFU/ml when irradiated (λ = 420 and 660 nm, 21.93 mW/cm^2^) with respect to log reduction in the dark (subtracted). Assays were performed three times in triplicate, and values shown are the mean ± standard error of the mean (SEM). ns is not statistically significant. Dark toxicity values are not shown but were subtracted for ZnTPP and Jeff-ZnTPP.

**Figure 8:**
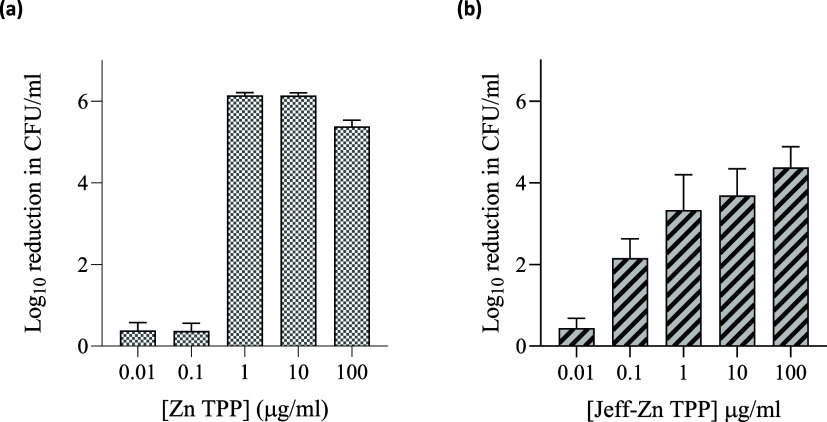
Dose–response for ZnTPP (a) and Jeff-ZnTPP (b). Data shown are the log reductions in CFU/mL when irradiated (λ = 420 and 660 nm, 21.93 mW/cm^2^) for 1 h with respect to DMSO controls. Assays were performed three times in triplicate and values shown are the mean ± SEM. Dark toxicity values are not shown but were subtracted for ZnTPP and Jeff-ZnTPP.

In initial experiments to establish the antimicrobial activity of porphyrin-polymers, the inactivation of *S. aureus* ATCC25923 by ZnTPP and Jeff-ZnTPP was negligible in the absence of light (<0.25 log reduction in CFU/mL). ZnTPP and the conjugated Jeff-ZnTPP polymer killed *S. aureus* when activated by irradiation at 420 nm, resulting in 0.95 log (ZnTPP) and 1.7 log (Jeff-ZnTPP) reduction in CFU/ml after 30 min, and irradiation for 60 min resulted in even greater killing of 5.4 and 6 log, respectively (Figure 7). Further investigation of the concentration dependence of *S. aureus* killing showed that for ZnTPP, concentrations from 1 to 100 μg/mL achieved 5–6 log killing whereas Jeff-ZnTPP showed a gradual increase in activity from 3 to 4 log over this concentration range (Figure 8). This pattern correlated with singlet oxygen yields, which were lower for the latter. Irradiation for 60 min, under the experimental conditions applied, had no significant effect on bacterial viability, as shown by comparison of the controls (*S. aureus* incubated in 10% DMSO) in dark and light conditions for 60 min (Figure S29).

**Figure 9:**
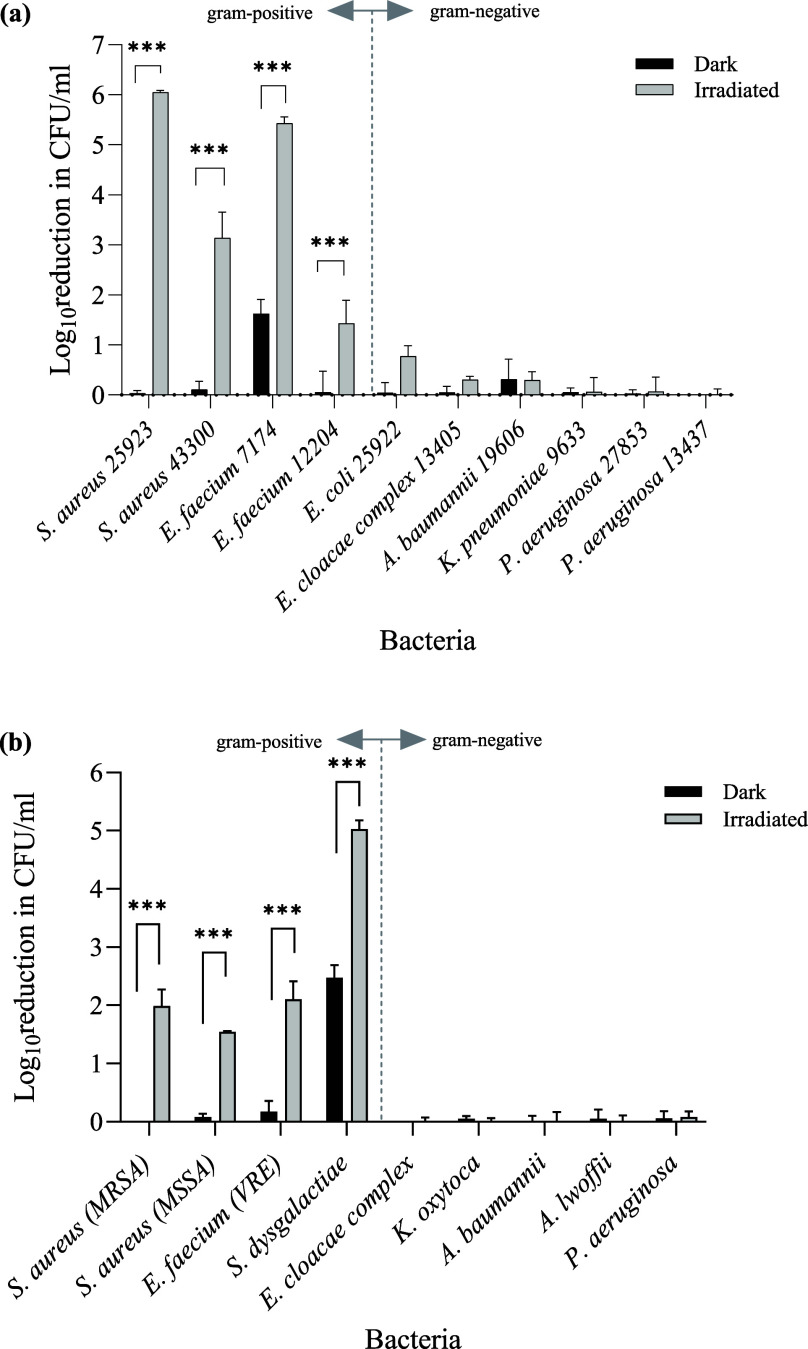
Antimicrobial spectrum of Jeff-ZnTPP using reference strains (a) or isolates recovered from wound infections (b). Data shown are the log reduction in CFU/mL when irradiated (λ = 420 and 660 nm, 21.93 mW/cm^2^) for 1 h with respect to DMSO controls. Assays were performed three times in triplicate and values shown are the mean ± SEM; *** denotes *P* ≤ 0.001.

The spectrum of activity of Jeff-ZnTPP was investigated further using antibiotic resistant and susceptible strains of Gram-positive and Gram-negative bacteria representing the ESKAPE pathogens and those associated with wound and device infections (Figure 9a). In addition, this polymer was tested against clinical isolates recovered directly from infected wounds (Figure 9b). Negligible activity toward Gram-negatives was found, and among Gram-positives, bactericidal activity varied (Figure 9). Selectivity for Gram-positives is a feature of several antibiotics and novel compounds. The outer cell membrane, composed of a phospholipid–lipopolysaccharide bilayer, which is unique to Gram-negatives reduces cell permeability or access to targets compared to Gram-positives, which lack this additional layer.^77^ A similar selectivity for Gram-positives has been shown with other porphyrin-based PDT. For example, Wang et al.^78^ showed selective bactericidal activity for *S. aureus* after PDT with positively charged porphyrins. Although they exhibit lower activity overall (<1 log), than we report here, the porphyrins TPPOH and TPPNH_2_, with electron-donating groups single bond NH_2_ and single bond OH, showed 30% killing of *S. aureus* and only 10% killing of *E. coli* when irradiated with light for 2 h at a porphyrin concentration of 9 μg/mL. Similarly, among a series of neutral and cationic tetraaryl-porphyrins, Banfi et al.^79^ reported generally greater PDT antimicrobial activity against Gram-positive bacteria (*S. aureus*) compared to Gram-negative bacteria (*E. coli* and *P. aeruginosa*), though with one, a dicationic porphyrin having potent activity against *P. aeruginosa*.

#### Biocompatibility Studies

**Figure 10:**
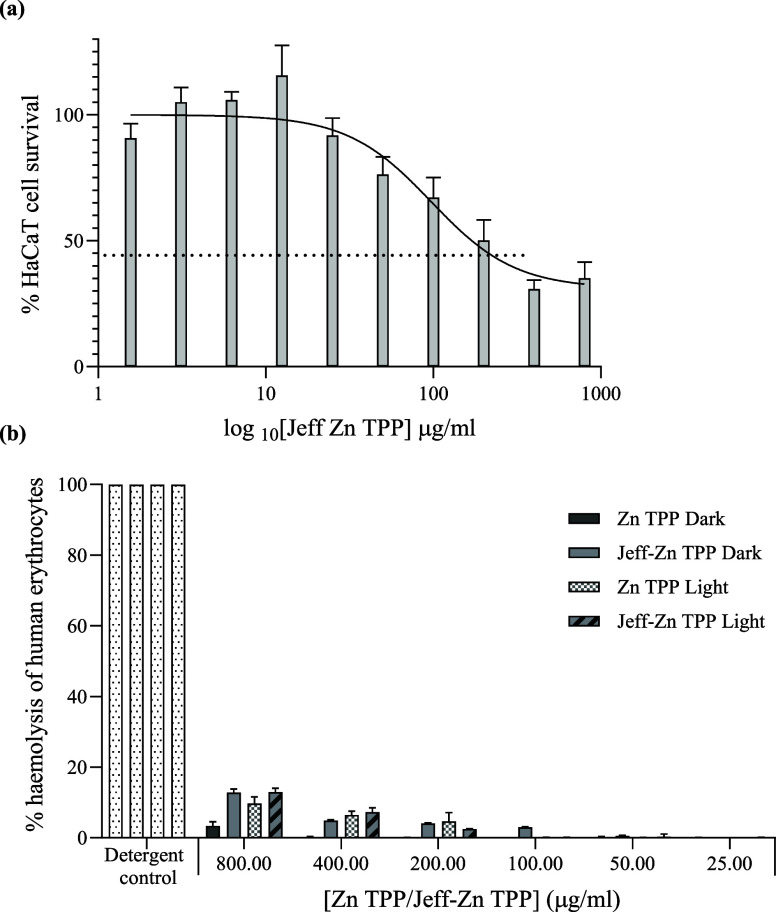
Biocompatibility investigations using cultured keratinocytes (a) and fresh human erythrocytes (b). Percentage survival of keratinocytes (approximately 3 × 10^5^ cells/mL) following 60 min incubation in the dark with Jeff-ZnTPP. Data are mean ± SEM based on the metabolic activity (MTT assay) for two separate assays in triplicate. The dashed line indicated 50% cell survival, and the best-fit curve for dose–response, from which IC_50_ was estimated, is shown (a). Percentage hemolysis of erythrocytes after incubation with ZnTPP or Jeff-ZnTPP for 60 min in the dark or irradiated (λ = 420 and 660 nm, 21.93 mW/cm^2^), relative to complete hemolysis using a Triton-X-100 detergent. Data are the mean ± SEM for three blood samples in duplicate.

When developing novel antimicrobial agents, selective toxicity to bacterial cells but limited damage to human cells is important. The concentration of Jeff-ZnTPP that demonstrated potent antimicrobial activity (100 μg/mL) resulted in the loss of viability of 22.8% of keratinocytes in culture. Lower concentrations were well-tolerated. An IC_50_ value of 169 μg/mL was estimated based on nonlinear regression (Figure 10a). Hemolysis of human erythrocytes was less than 3% for ZnTPP and Jeff-ZnTPP at 100 μg/mL, and even with irradiation, treatment was well-tolerated by these cells up to 800 μg/mL, with less than 13% hemolysis observed (Figure 10b).

## Conclusions

The effect of incorporating porphyrin photosensitizing units onto a polymer backbone has been thoroughly studied. On the femtosecond–nanosecond time scales, there are few differences between the reference compound, ZnTPP, and the Jeff-ZnTPP polymer, indicating that incorporation of the porphyrin into the polymer does not hinder/detract from the inherent photophysical properties of the porphyrin system. However, on the microsecond time scale, we observe an enhanced long-lived triplet excited state, with a significantly increased lifetime, ca. 72 μs (in THF), as compared to 30 μs for ZnTPP. The polymer encapsulates the photosensitizing units, which acts as a shield against its local environment and makes the porphyrin less prone to quenching, and thus the longer-lived triplet state. A similar effect was observed by Issberner et al.^80^ who incorporated dendrimer branches onto a ruthenium moiety. They noted an increase in the triplet lifetime of the system (1.7 μs as opposed to 990 ns) due to the shielding effect induced by encapsulating the photosensitizer unit in the dendrimers. Similarly, Brady et al.^46^ bound two different porphyrins to copolymers of 2-hydroxy methacrylate with methacrylic acid or 2-(diethylamino)ethyl methacrylate and observed an increased triplet lifetime of 230/1145 μs, respectively, further highlighting the beneficial effect of incorporating photosensitizers into large polymeric assemblies.

In addition, there were significant binding interactions between Jeff-ZnTPP polymer and biological molecules, DNA and serum albumin, compared to ZnTPP alone. UV–vis absorption and fluorescence quenching studies confirmed that Jeff-ZnTPP exhibits stronger DNA intercalation than ZnTPP. Similarly, Jeff-ZnTPP showed greater affinity toward BSA, forming more stable complexes than ZnTPP. These enhanced binding properties of Jeff-ZnTPP are likely due to its increased surface area and flexible conformation. While more specific investigation of bacterial DNA and localized protein interactions with Jeff-ZnTPP are warranted to support the photokilling mechanism and targets involved, the interaction profiles of Jeff-ZnTPP shown here highlight its potential applications, more broadly in DNA sensing, drug delivery, and other applications of photodynamic therapy (e.g., cancer therapeutics). Further investigations are warranted to fully elucidate the mechanisms underlying these enhanced interactions.

In terms of its antimicrobial activity, the novel porphyrin-polymer appeared to be selective for Gram-positive bacteria with poor activity against the Gram-negative bacteria tested, and this was found for reference strains and clinical isolates recovered directly from wound infections. This narrower antimicrobial spectrum is of potential benefit for infections in which the etiology favors Gram-positives, such as wound infections and device-related infections, where Staphylococci and Streptococci are the more commonly found causative agents.^81^ Favorable biocompatibility was evidenced for Jeff-ZnTPP based on low toxicity to human skin cells (cultured keratinocytes) and red-blood cells (primary erythrocytes) and highlights its potential for progression to in vivo investigation. Jeff-ZnTPP’s potent Gram-positive activity has been demonstrated and dynamic interaction with biological targets have been elucidated, shedding light on its efficacy and potential safety profile. Moving forward, further exploration of porphyrin–polymer complexes promises to unlock new possibilities in antimicrobial therapy and biomedical research, ultimately improving patient outcomes and advancing the field of photomedicine.

In summary, the utilization of Jeff-ZnTPP, a new porphyrin–polymer, as a photosensitizer prodrug in aPDT offers a novel approach to combating microbial pathogens. While more specific investigation of bacterial DNA and protein interactions with Jeff-ZnTPP are warranted to support the photokilling mechanism and targets involved, its binding to serum proteins is also important in terms of other therapeutic and diagnostic aspects.

## Supporting Information Available

Synthetic route to Zn-COOH-TPP (Scheme S1) as well as ^1^H and ^13^C NMR spectra of Me-pcTPP, Zn-Me-pcTPP, Zn-COOH-TPP, and Jeffamine ED-900 polymer; ESI-MS of (Zn-COOH-TPP), FT-IR spectrum of Jeffamin,e and Jeff-ZnTPP polymer; UV–vis spectra of Me-pcTPP, Zn-Me-pcTPP, and Zn-COOH-TPP; GPC RI traces of Jeffamine and Jeff-ZnTPP polymers (Figure S16 and Table S1); UV–vis spectrum of Jeff-ZnTPP in 10% dimethyl sulfoxide (DMSO) (v/v in PBS) at 37 °C during 72 h, UV–vis spectrum of Jeff-ZnTPP by light irradiation at 590 nm in THF; time-dependent absorption spectra at 415 nm of DPBF (in DMF) in the presence of ZnTPP, and Jeff-ZnTPP polymer; spectral transient absorption profile for Jeff-ZnTPP in ethyl acetate over a 1500 ps time range (Figure S20); fluorescence decay profile of Jeff-ZnTPP (λ_ex_ = 375 nm) in ethyl acetate over a 35 ns time range (Figure S21); spectral transient absorption profile for Jeff-ZnTPP in ethyl acetate over a 500 μs time range (Figure S22); absorption spectra of ZnTPP and in Tris-HCl buffer upon addition of CT DNA (Figure S23); fluorescence quenching curves of EB bound to DNA in the presence of ZnTPP in Tris-HCl buffer (Figure S24); UV–vis absorption and fluorescence quenching curves of BSA in PBS solution in the presence of different amounts of ZnTPP (Figures S25 and S26); scatchard plot of the fluorescence titrations of (a) Jeff-ZnTPP polymer and (b) ZnTPP with BSA (Figure S27); antimicrobial activity of porphyrins against *E. coli* (Figure S28); comparison of the number of bacteria (*S. aureus* 25923) remaining following incubation in 10% DMSO, 90% PBS in the dark for 60 min vs or irradiated with light for 60 min (Figure S29); and bacterial strains and isolates used in the present study (Table S2) (PDF)
