# Supplementary material for: Porphyrin-Polymer as a Photosensitizer Prodrug for Antimicrobial Photodynamic Therapy and Biomolecule Binding Ability
Source: Biomacromolecules. 2024 Nov 8;25(12):7736–49. doi: 10.1021/acs.biomac.4c01011 (PMC11632779; doi:10.1021/acs.biomac.4c01011)
Supplement: Supplementary file 1 — bm4c01011_si_001.pdf [file bm4c01011_si_001.pdf]

A porphyrin-polymer as a photosensitizer prodrug for antimicrobial photodynamic therapy and biomolecule binding ability

Leila Tabrizi[1,$], Ross McGarry[1,$], Kaja Turzanska[2], Lazaros Varvarezos[3,4], Muireann Fallon[2], Ruairi Brannigan[1], John T. Costello[3], Deirdre Fitzgerald-Hughes[2*], Mary T. Pryce[1*]

[1] School of Chemical Sciences, Dublin City University, Dublin, D09W6Y4, Ireland
[2] Clinical Microbiology, Royal College of Surgeons in Ireland, RCSI Education and Research, Beaumont Hospital, Beaumont, Dublin, D09YD60, Ireland
[3] School of Physical Sciences, Dublin City University, Dublin 9, D09 K2WA, Ireland
[4] Department of Physics, University of Ioannina, GR-45110 Ioannina, Greece

$ joint authors

*Email: mary.pryce@dcu.ie

*Email: dfitzgeraldhughes@rcsi.ie

**Contents**	Pages
Scheme S1. Synthetic route to Zn-COOH-TPP, **3**	3
Figure S1. ^{1}HNMR spectrum of Me-pcTPP in $CDCl_3$	4
Figure S2.^{13}CNMR spectrum of Me-pcTPP in $CDCl_3$	5
Figure S3.^{1}HNMR spectrum of Zn-Me-pcTPP, 2 in $CDCl_3$.	6
Figure S4.^{13}CNMR spectrum of Zn-Me-pcTPP, 2 in $CDCl_3$.	7
Figure S5.^{1}H NMR spectrum of (Zn-COOH-TPP), 3 in DMSO-d_6.	8
Figure S6.^{13}C NMR spectrum of (Zn-COOH-TPP), 3 in DMSO-d_6.	9
Figure S7. ESI-MS of (Zn-COOH-TPP), 3, in acetonitrile.	10
Figure S8.^{1}H NMR spectrum of Jeffamine ED-900 polymer in $CDCl_3$.	11
Figure S9.^{1}H NMR spectrum of Jeff-Zn TPP polymer in $CDCl_3$.	11
Figure S10. FT-IR spectrum of Jeffamine ED-900 polymer	12
Figure S11. FT-IR spectrum of Jeff-Zn TPP polymer	12
Figure S12. FT-IR spectra of Jeff-Zn TPP and Jeff polymers in chloroform.	13
Figure S13. UV-vis spectrum of Me-pcTPP in CH_2Cl_2	14
Figure S14. UV-Vis spectrum of Zn-Me-pcTPP, 2 in CH_2Cl_2.	14
Figure S15. UV-Vis spectrum of (Zn-COOH-TPP), 3 in THF.	15

Figure S16. GPC RI traces of Jeffamine and Jeff-Zn TPP polymers	16
Table S1. GPC results of Jeff-Zn TPP polymers	16
Figure S17. UV-Vis spectrum of Jeff-Zn TPP in 10% dimethyl sulfoxide (DMSO) (v/v in PBS) at 37 ºC during 72 h.	17
Figure S18. UV-Vis spectrum of Jeff-Zn TPP by light irradiation at 590 nm in THF.	17
Figure S19. Time-dependent absorption spectra at 415 nm of DPBF (in DMF) in the presence of Zn-TPP, and Jeff-Zn TPP polymer. Insert: The decay rate of the absorption of DPBF at 415 nm during illumination of Zn- Zn-TPP, and Jeff-Zn TPP polymer.	18
Figure S20. The spectral transient absorption profile for Jeff-Zn TPP in Ethyl acetate over a 1500 ps time range, with the insert displaying the kinetic time traces for Jeff-Zn TPP recorded at 450 nm (red datapoints) and 650 nm (blue datapoints), displayed over 50 ps.	19
Figure S21. Fluorescence decay profile of Jeff-Zn TPP (λ_{ex} = 375 nm) in ethyl acetate over a 35 ns time range. The red solid curve represents the fitted curve with the insert displaying the emission spectrum.	20
Figure S22. The spectral transient absorption profile for Jeff-Zn TPP in Ethyl Acetate over a 500 µs time range with the insert displaying the kinetic time trace for Jeff-Zn TPP recorded at 550 nm in Ethyl Acetate, recorded over a 500 µs time range.	21
Figure S23. Absorption spectra of Zn TPP (10 µM) and in Tris-HCl buffer upon addition of CT DNA (0-24 µM). Arrow shows that the absorption intensities decrease upon increasing DNA concentration. Insert: Plot of [DNA] / (ε_a - ε_f) versus [DNA] for the titration of the Zn TPP and with CT DNA.	22
Figure S24. Fluorescence quenching curves of EB bound to DNA in the presence of Zn TPP in Tris-HCl buffer. [DNA] = 5 µM, [EB] = 5 µM and [compounds] = 0-24 µM. Insert: Stern–Volmer plot of fluorescence titrations of Zn TPP with CT DNA.	23
Figure S25. UV-vis absorption of BSA (10 µM) in PBS solution in the presence of different amounts (0-24 µM) of Zn TPP.	24
Figure S26. Fluorescence quenching curves of BSA (10 µM) in PBS solution in the presence of different amounts (0-24 µM) of Zn TPP. Insert: Stern–Volmer plot of the fluorescence titrations.	25
Figure S27. Scatchard plot of the fluorescence titrations of (a) Jeff-Zn TPP polymer and (b) Zn TPP (0-24 µM) with BSA (10 µM).	25
Figure S28. Antimicrobial activity of porphyrins against *E.coli*.	26
Figure S29. Effect of 60 min irradiation on *S. aureus*	27

Table S2. Bacterial strains and isolates used in the present study	28-29

Scheme of synthesis

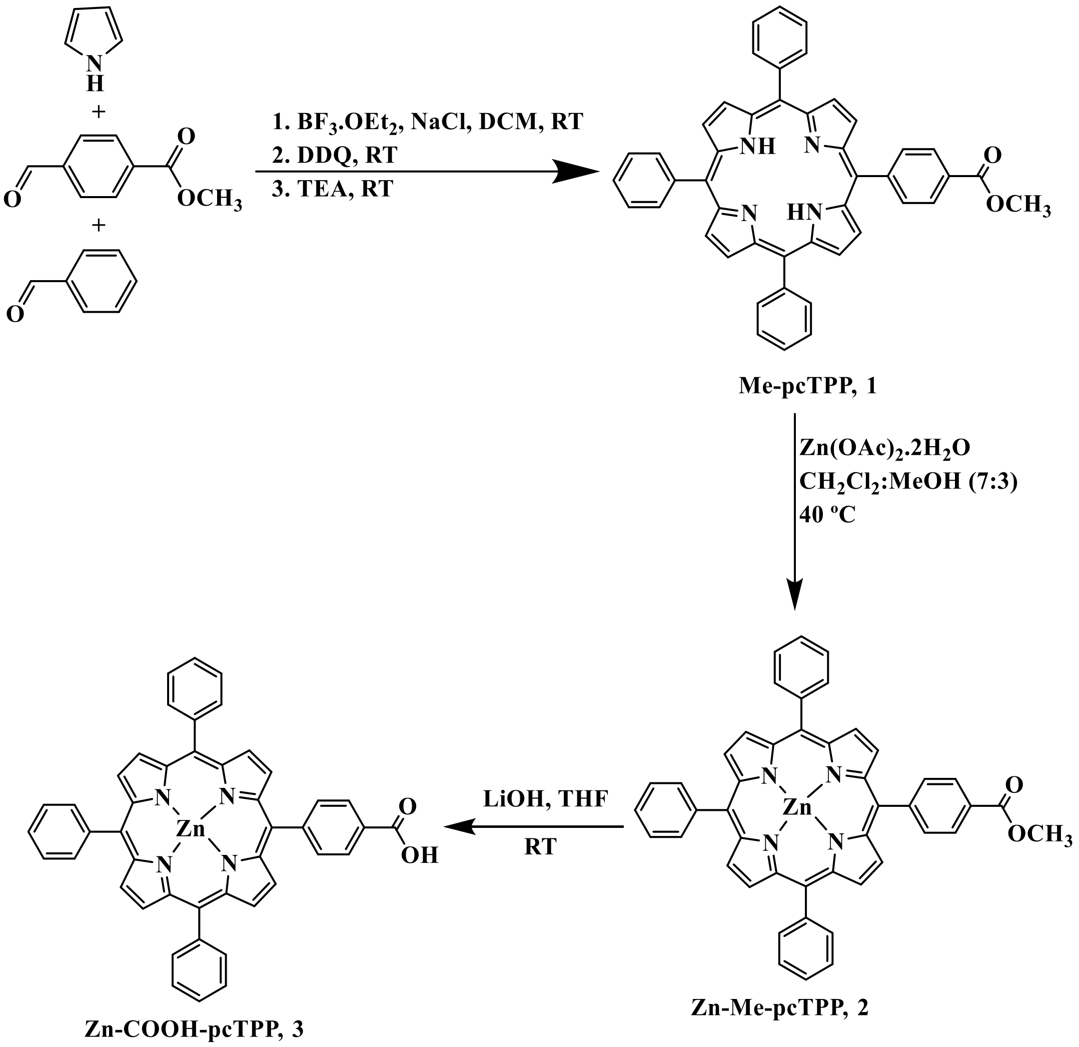

Scheme S1. Synthetic route to Zn-COOH-TPP, **3**

Characterizations

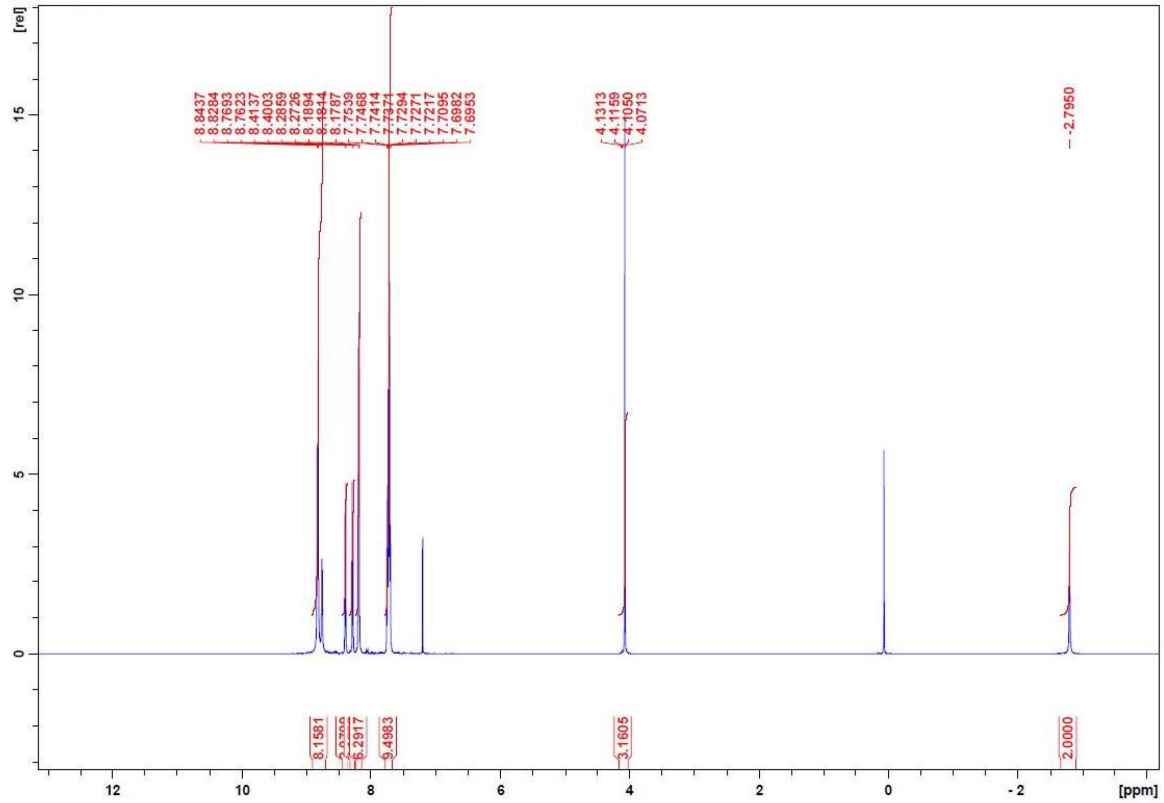

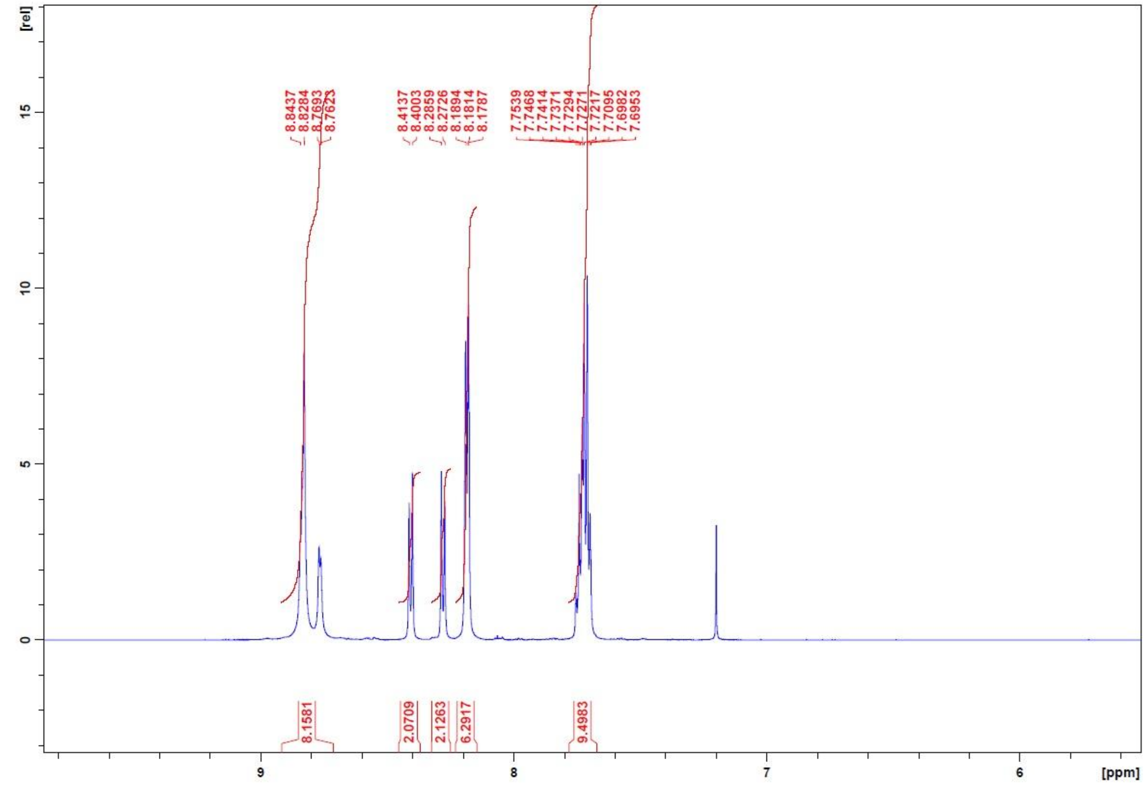

Figure S1. ^{1}HNMR spectrum of Me-pcTPP in $CDCl_3$

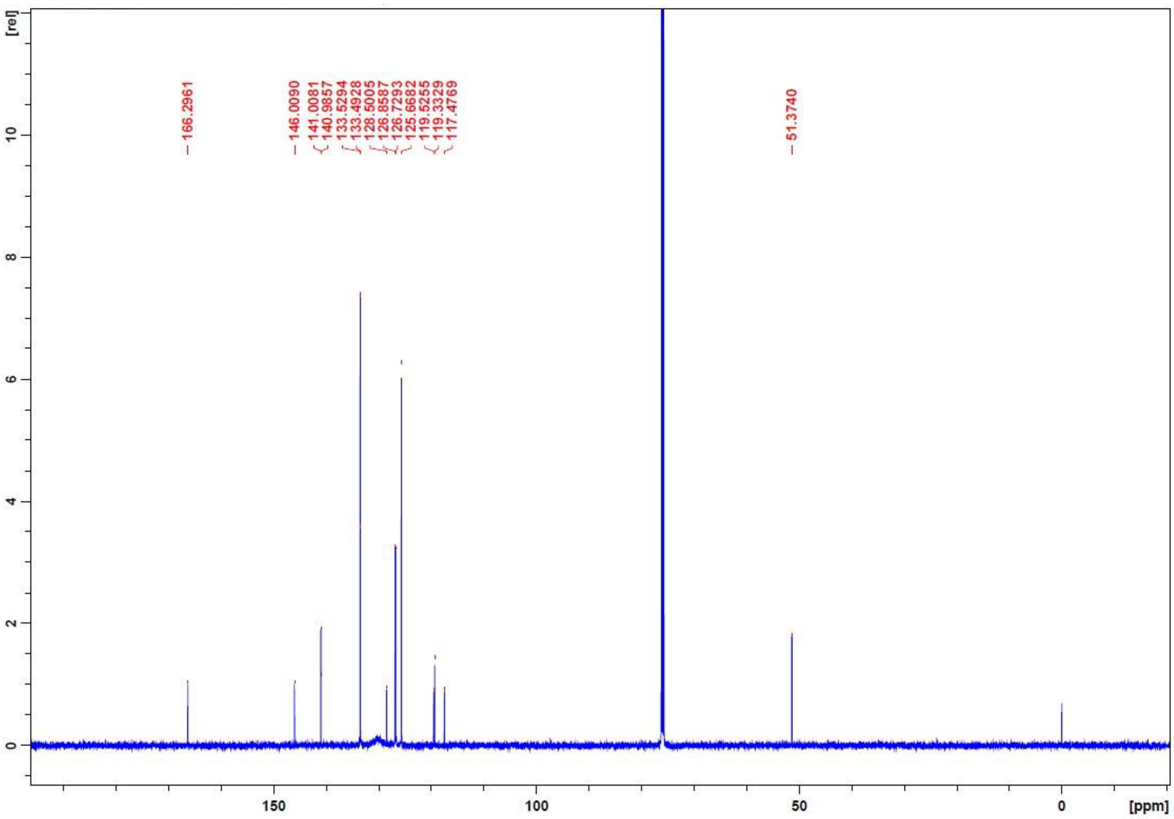

Figure S2. ^{13}C NMR spectrum of Me-pcTPP in $CDCl_3$

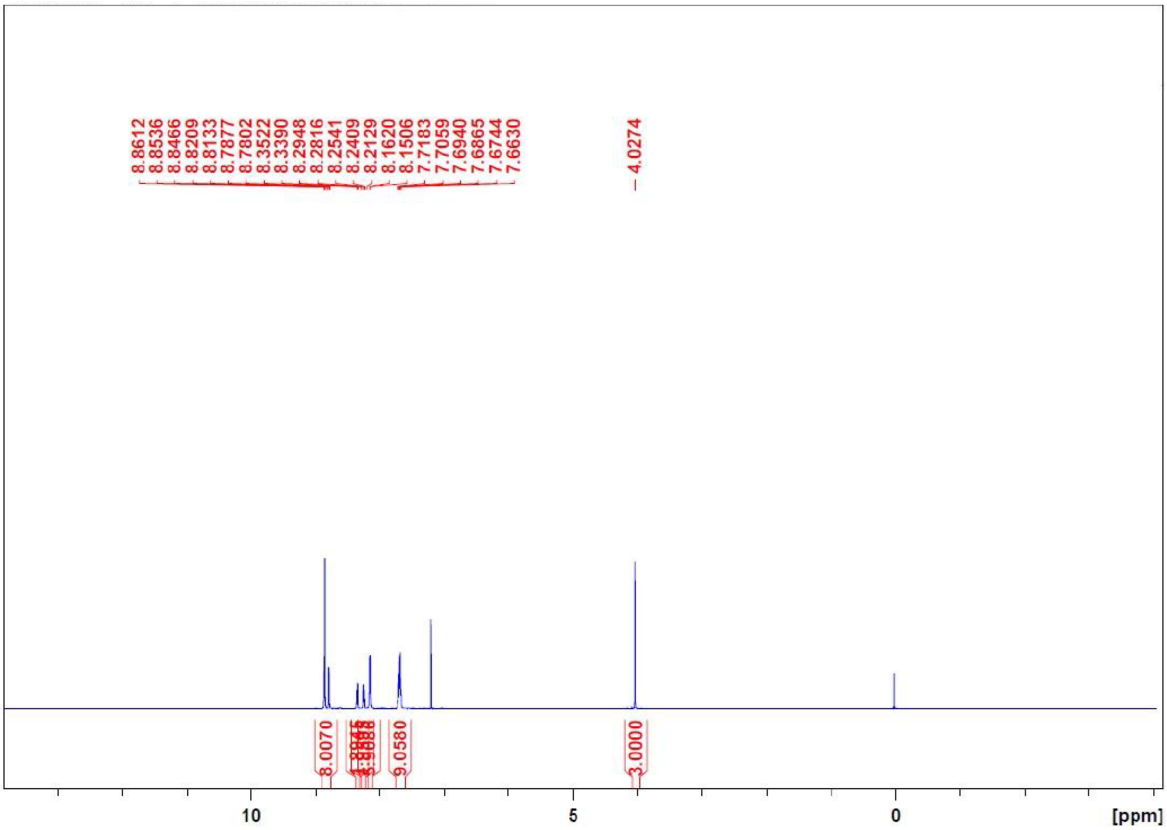

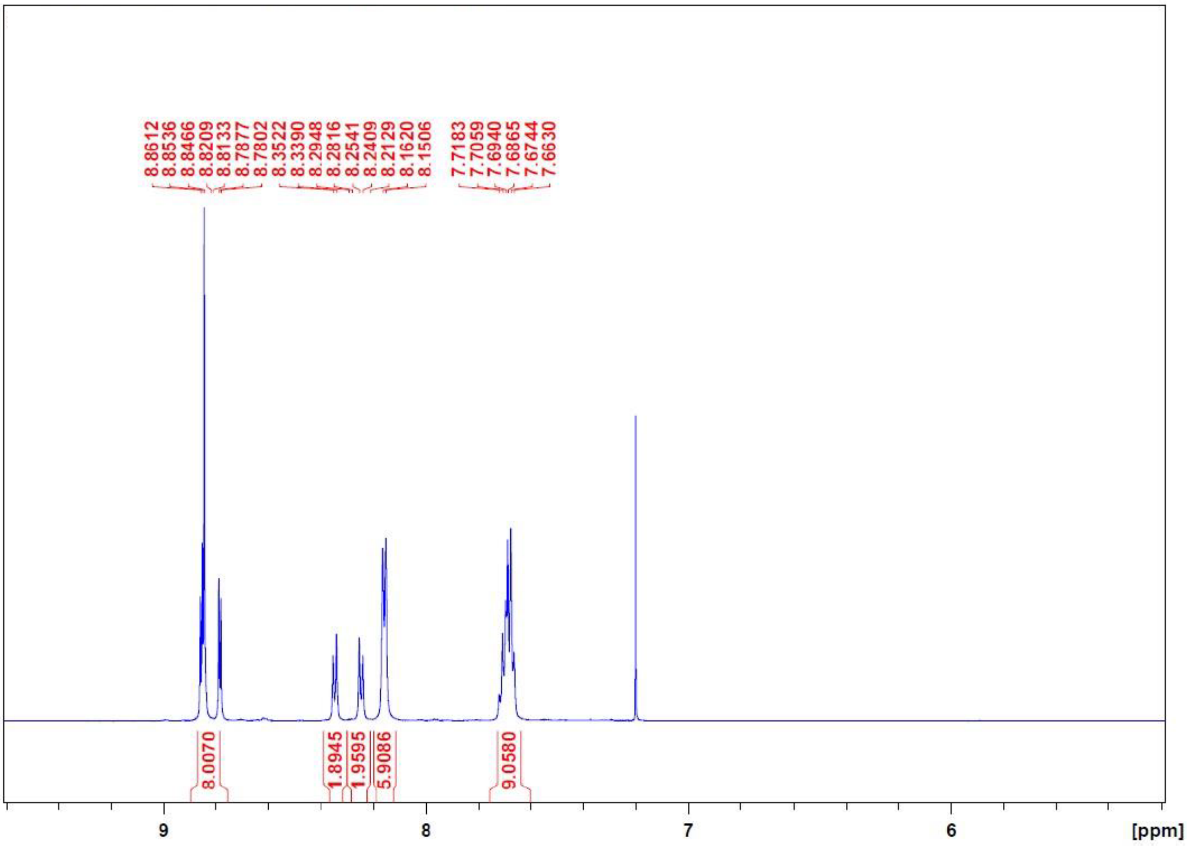

Figure S3.^{1}HNMR spectrum of Zn-Me-pcTPP (**2)** in $CDCl_3$.

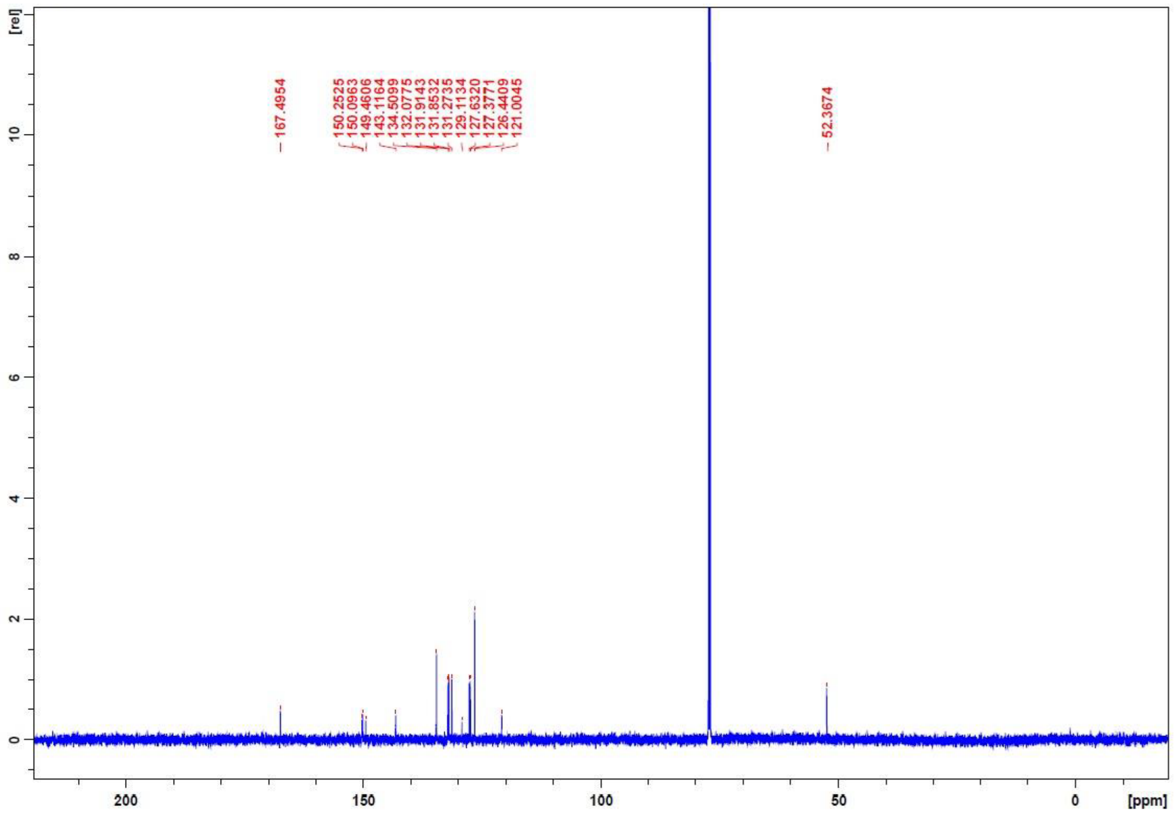

Figure S4.^{13}CNMR spectrum of Zn-Me-pcTPP (**2)** in $CDCl_3$.

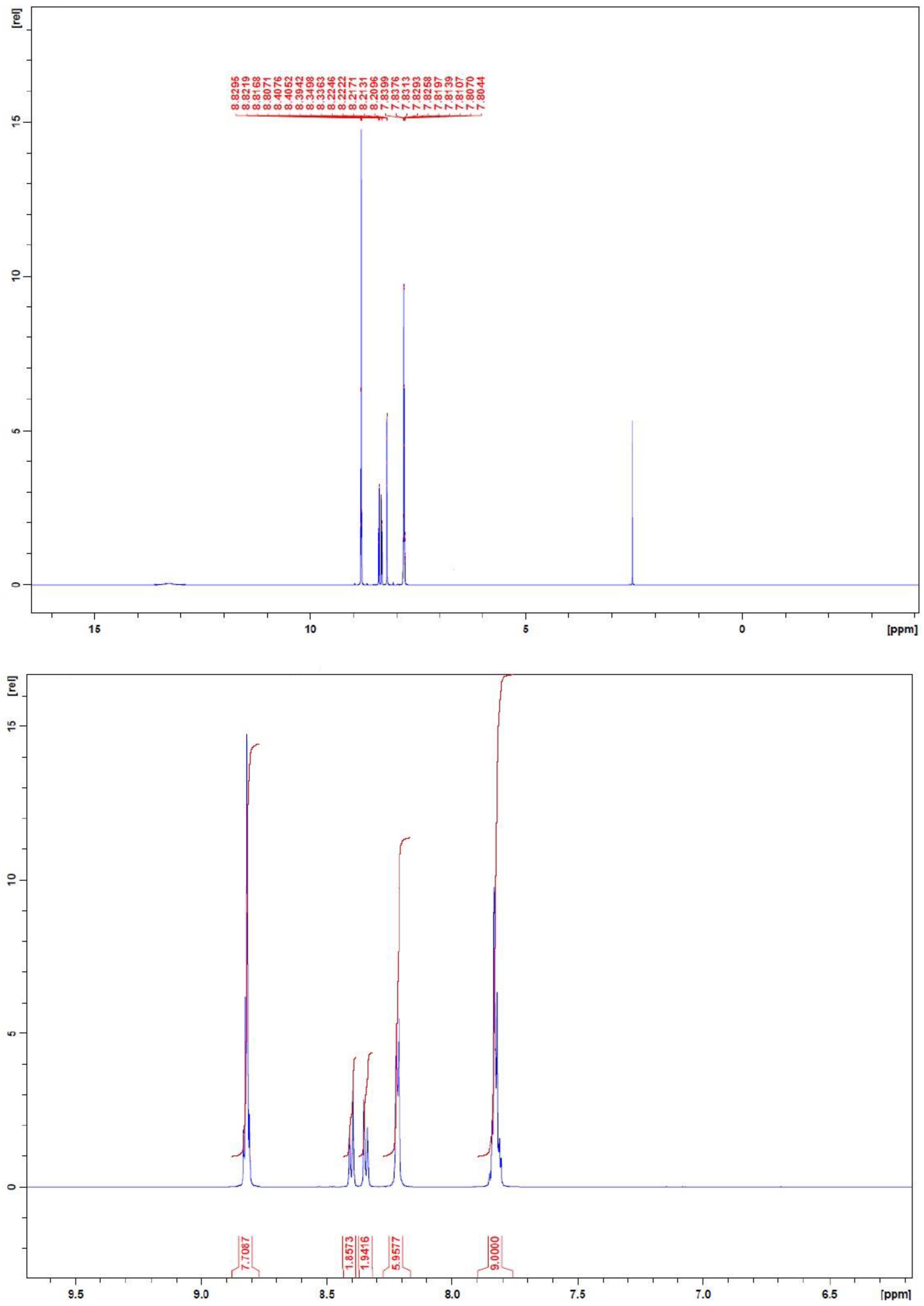

Figure S5.^{1}H NMR spectrum of (Zn-COOH-TPP), (**3)** in DMSO-d_6.

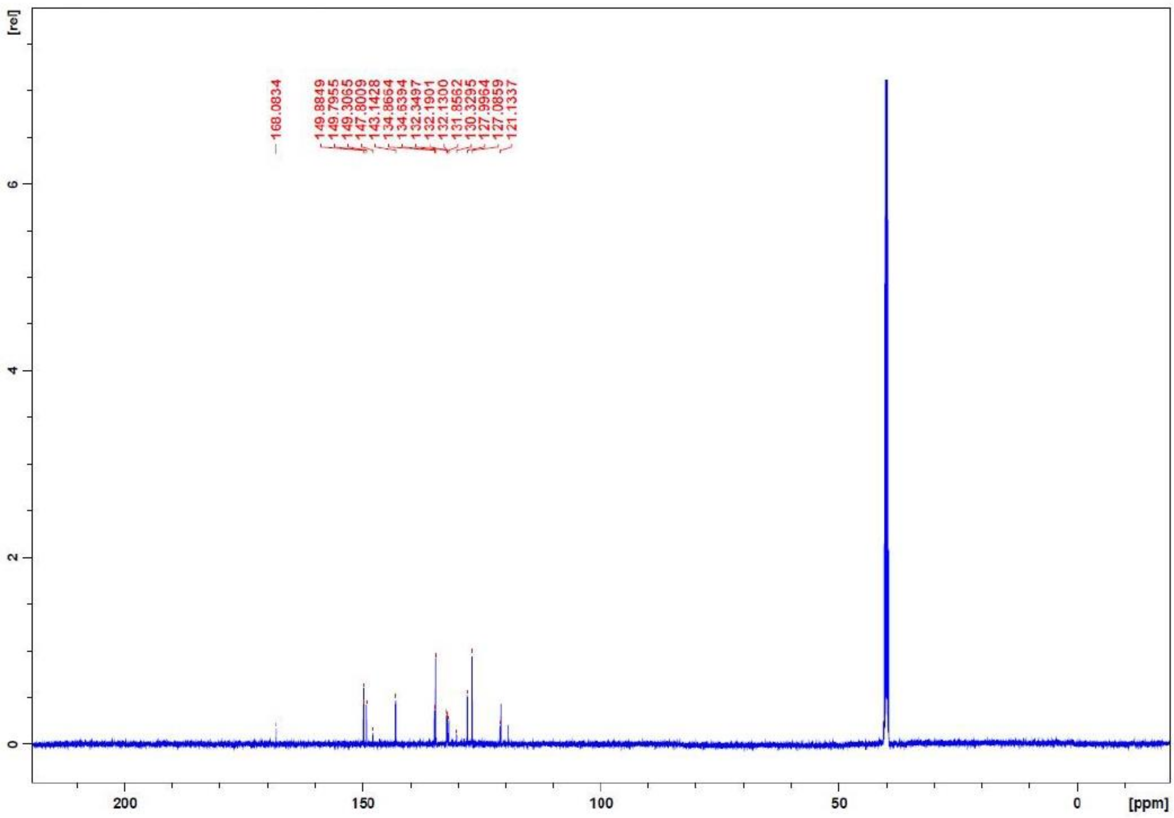

Figure S6.^{13}C NMR spectrum of (Zn-COOH-TPP), (**3)** in DMSO-d_6.

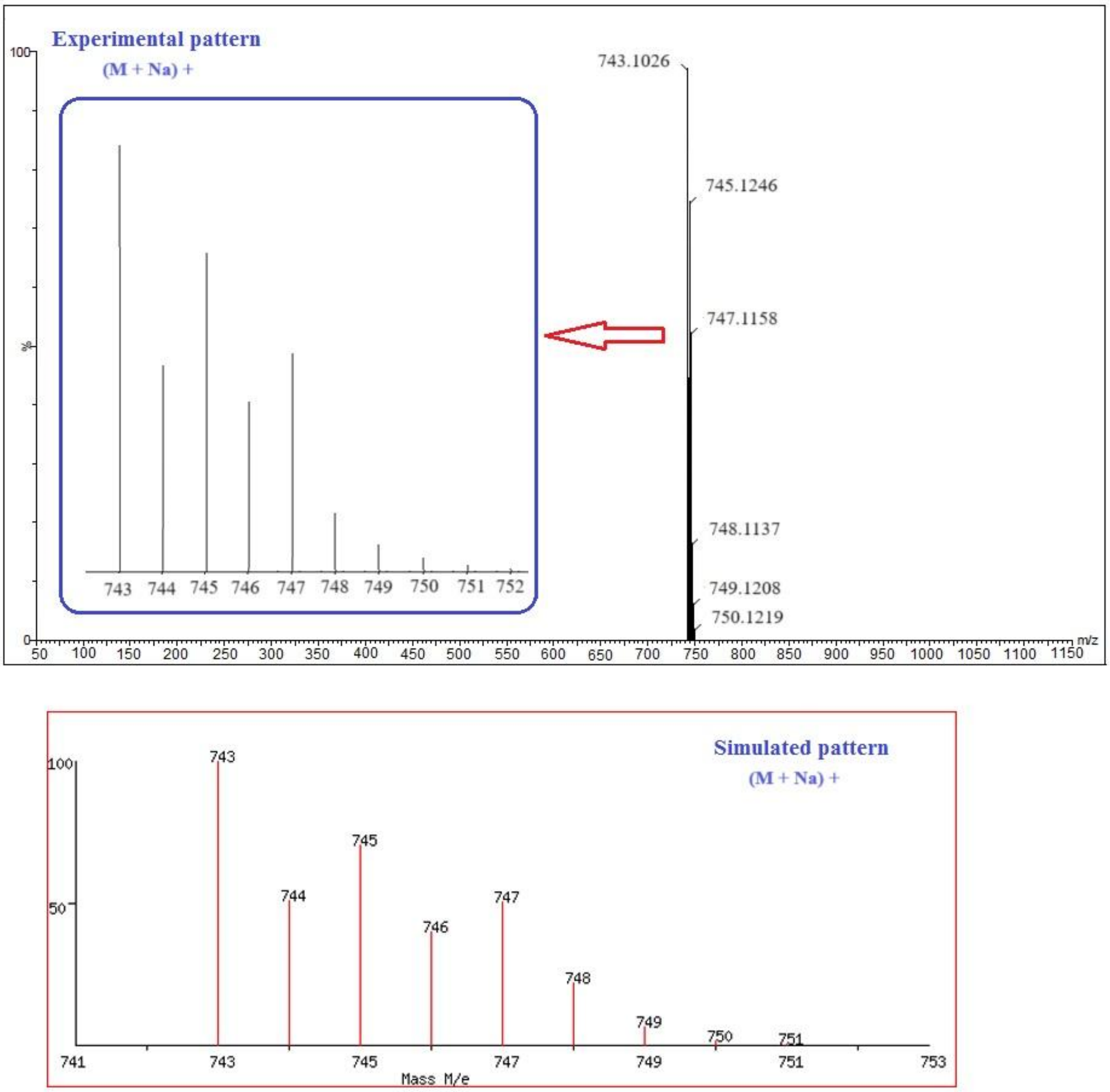

Figure S7. ESI-MS of (Zn-COOH-TPP), (**3**), in acetonitrile

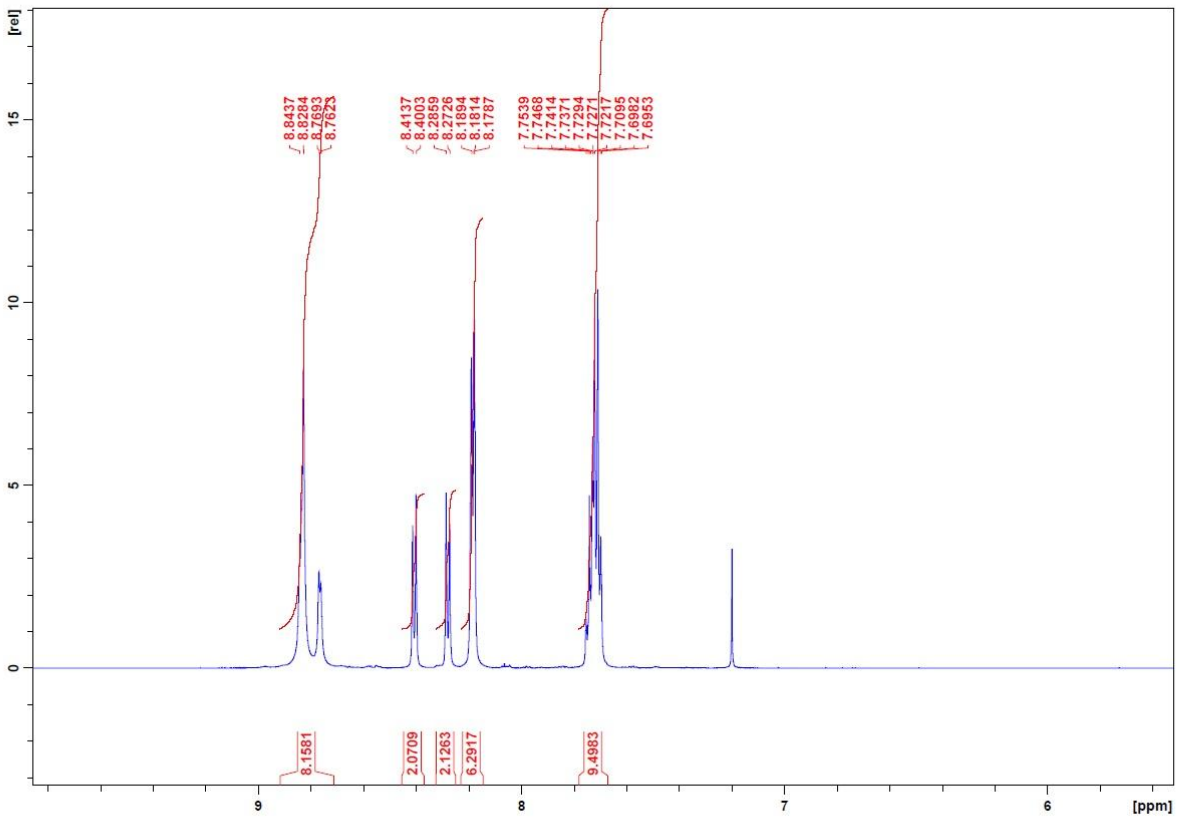

Figure S8.^{1}H NMR spectrum of Jeffamine ED-900 polymer in $CDCl_3$.

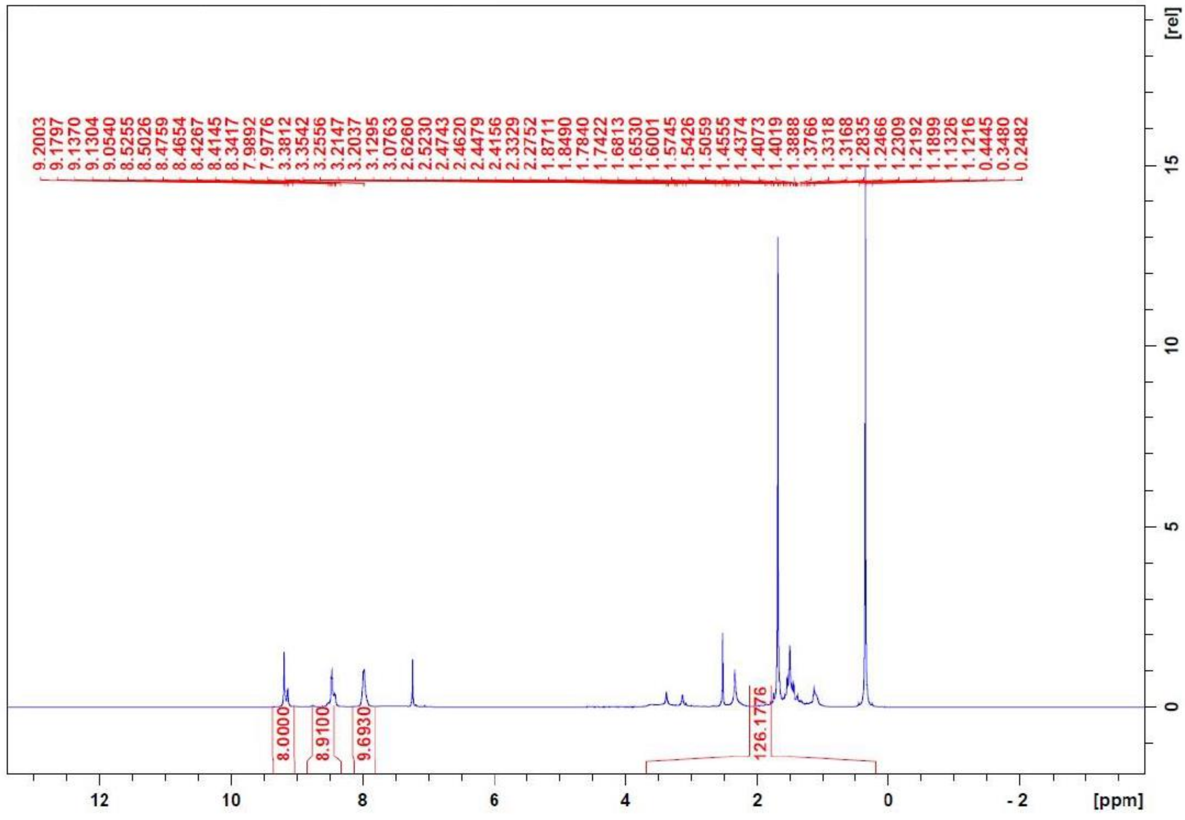

Figure S9.^{1}H NMR spectrum of Jeff-ZnTPP polymer in $CDCl_3$.

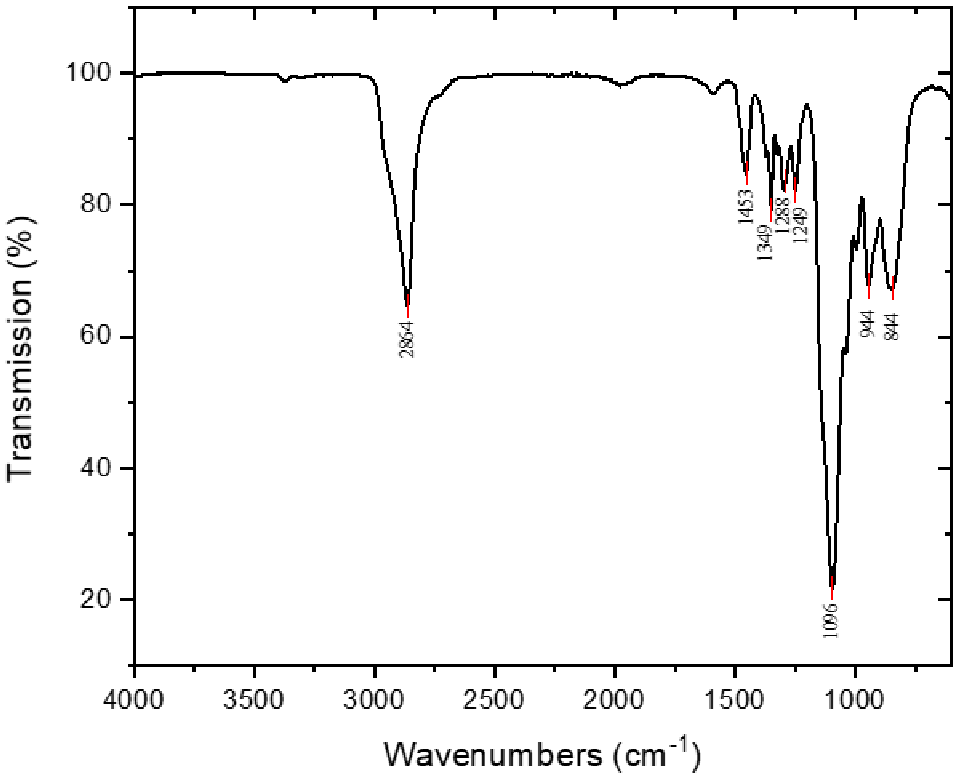

Figure S10. FT-IR spectrum of Jeffamine ED-900 polymer.

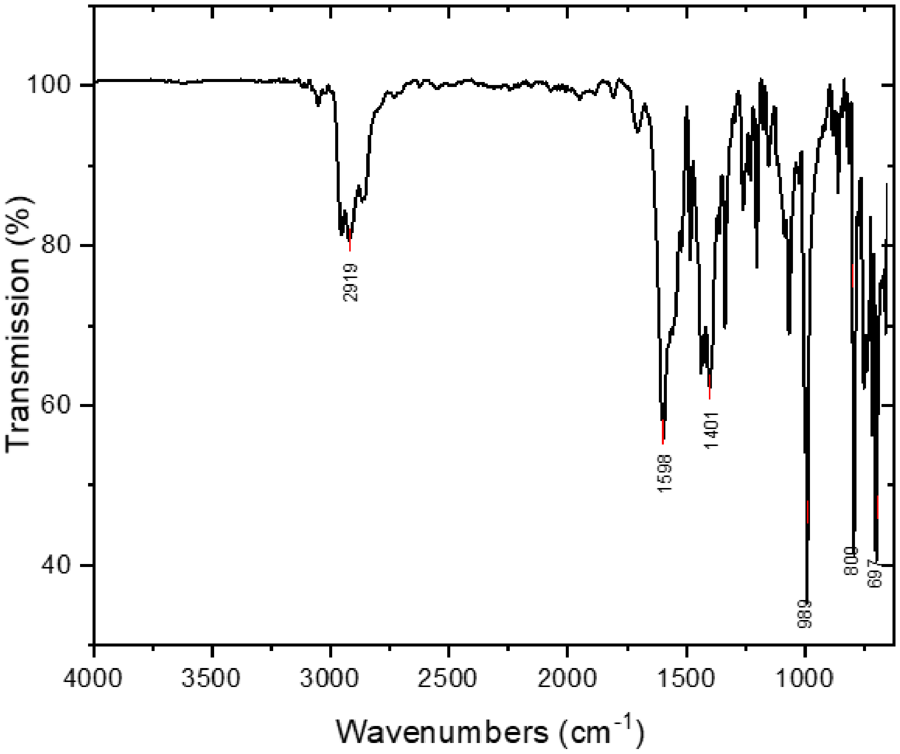

Figure S11. FT-IR spectrum of Jeff-ZnTPP polymer.

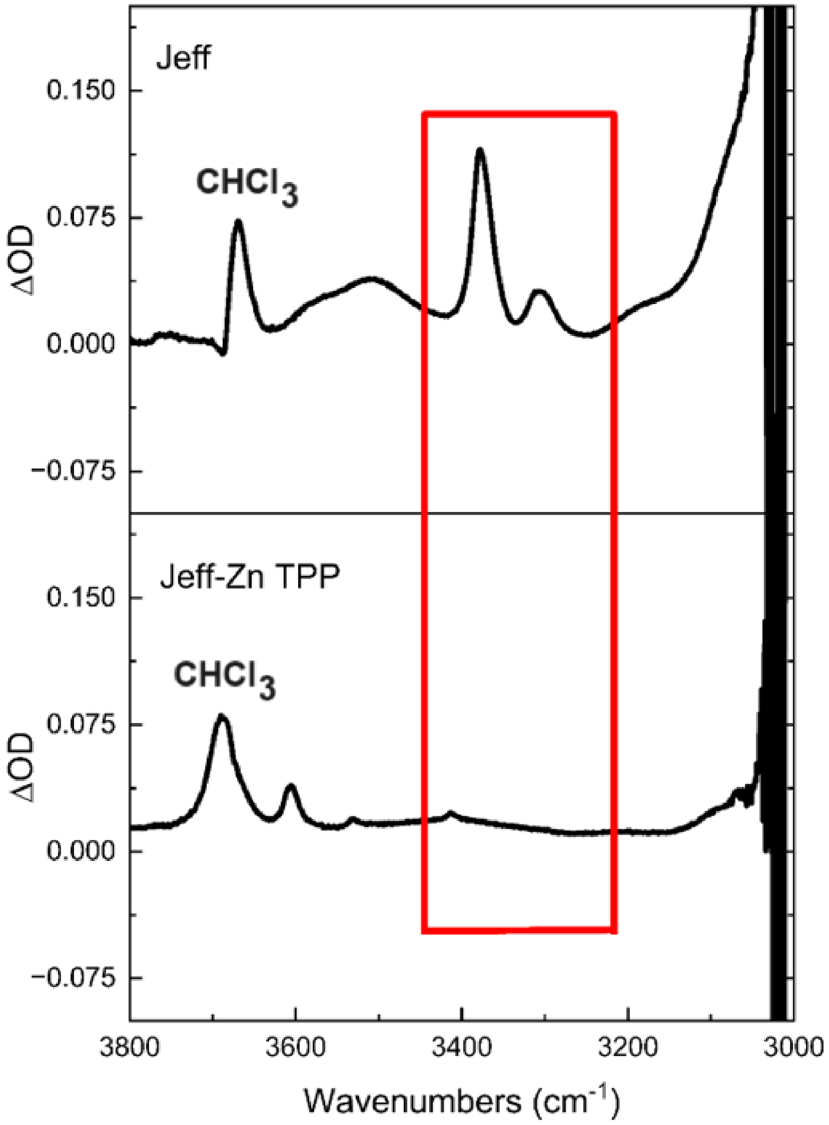

Figure S12. FT-IR spectra of the Jeff-ZnTPP and Jeff polymers in chloroform.

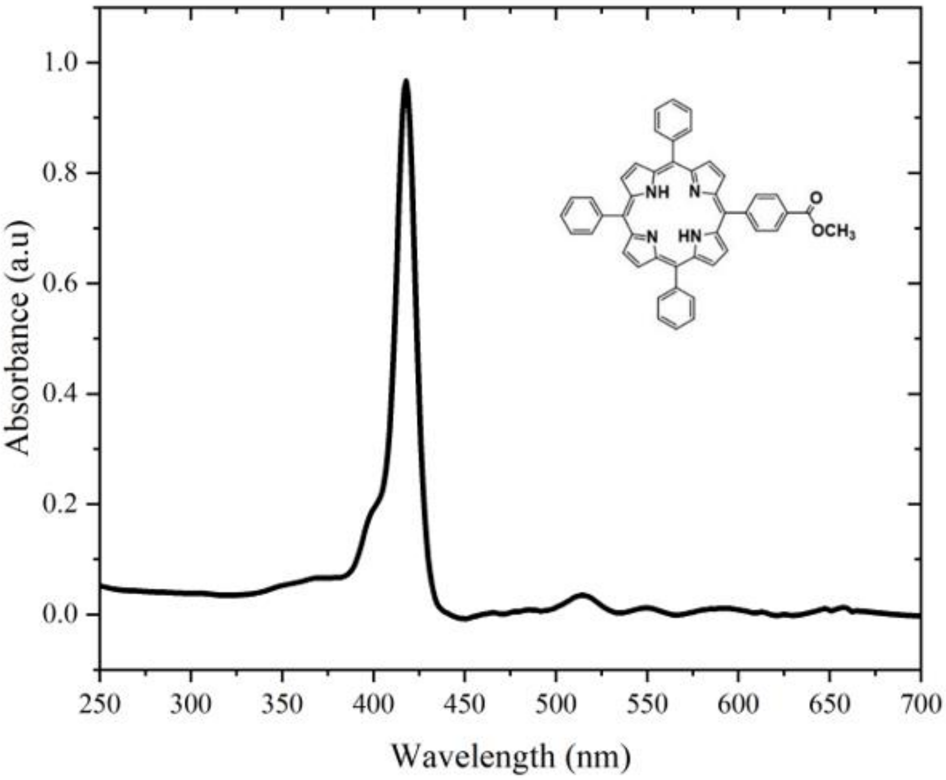

Figure S13. UV-vis spectrum of Me-pcTPP in CH_2Cl_2

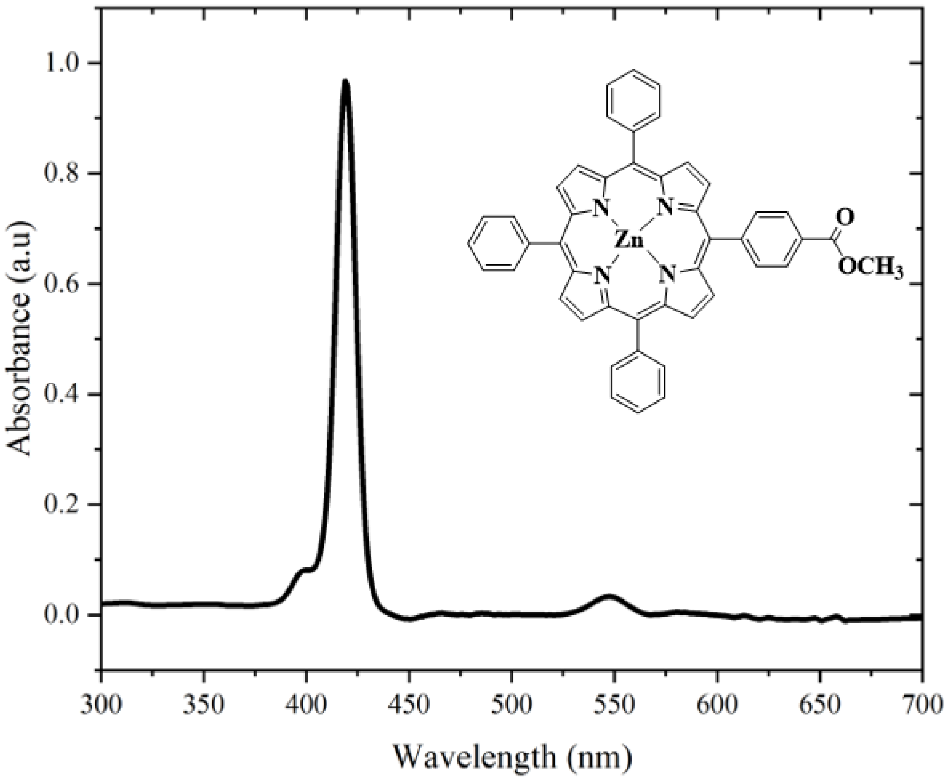

Figure S14. UV-Vis spectrum of Zn-Me-pcTPP, (**2)** in CH_2Cl_2.

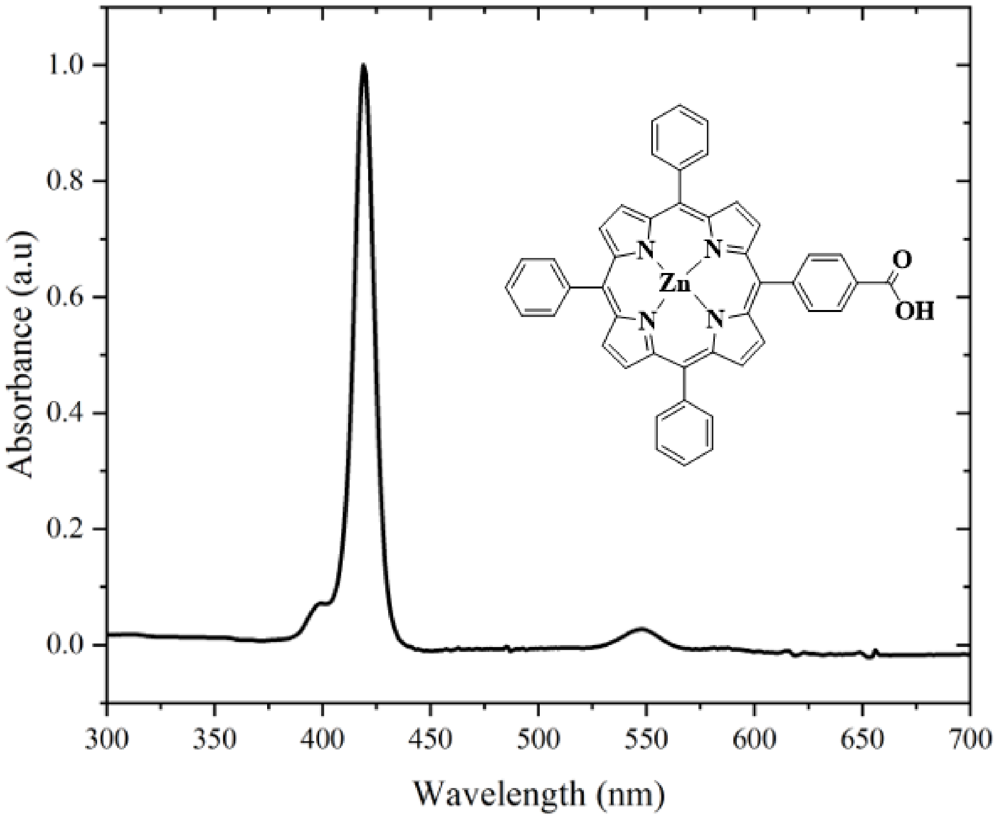

Figure S15. UV-Vis spectrum of (Zn-COOH-TPP), (**3)** in THF.

GPC results

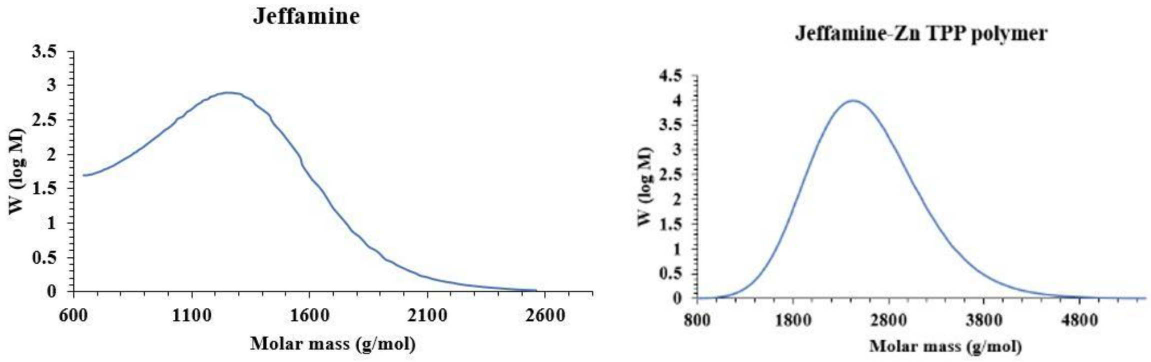

Figure S16. GPC RI traces of Jeffamine and Jeff-ZnTPP polymers.

Table S1. GPC results of Jeff-ZnTPP polymers

	Jeffamine	Jeff-Zn TPP
M_n	1061	2310 g/mol
M_w	1154	2442 g/mol
M_p	1266	2436 g/mol
$Đ_M$	1.08	1.06

Stability of polymer

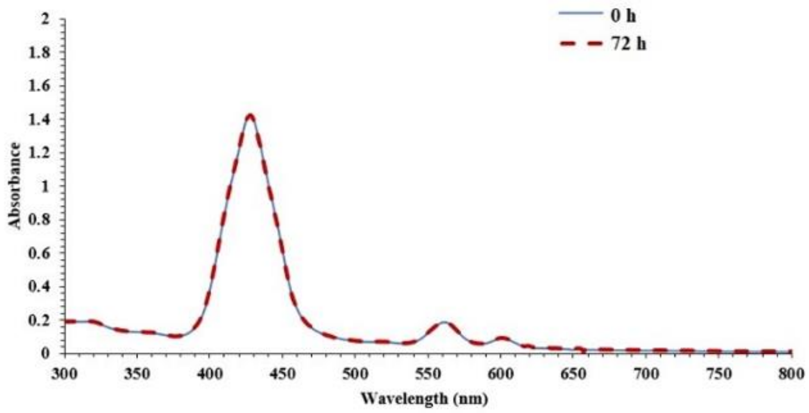

Figure S17. UV-Vis spectrum of Jeff-Zn TPP in 10% dimethyl sulfoxide (DMSO) (v/v in PBS) at 37 ºC after 72 h.

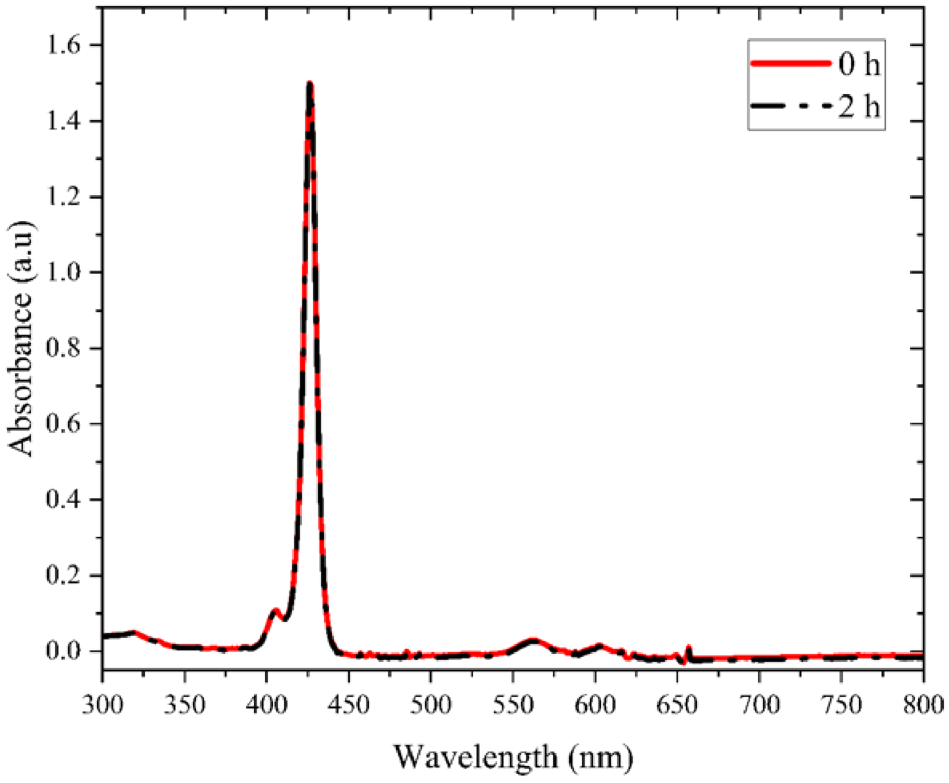

Figure S18. UV-Vis spectrum of Jeff-Zn TPP by light irradiation at 590 nm in THF.

Singlet Oxygen ($^1O_2$) Production Measurements

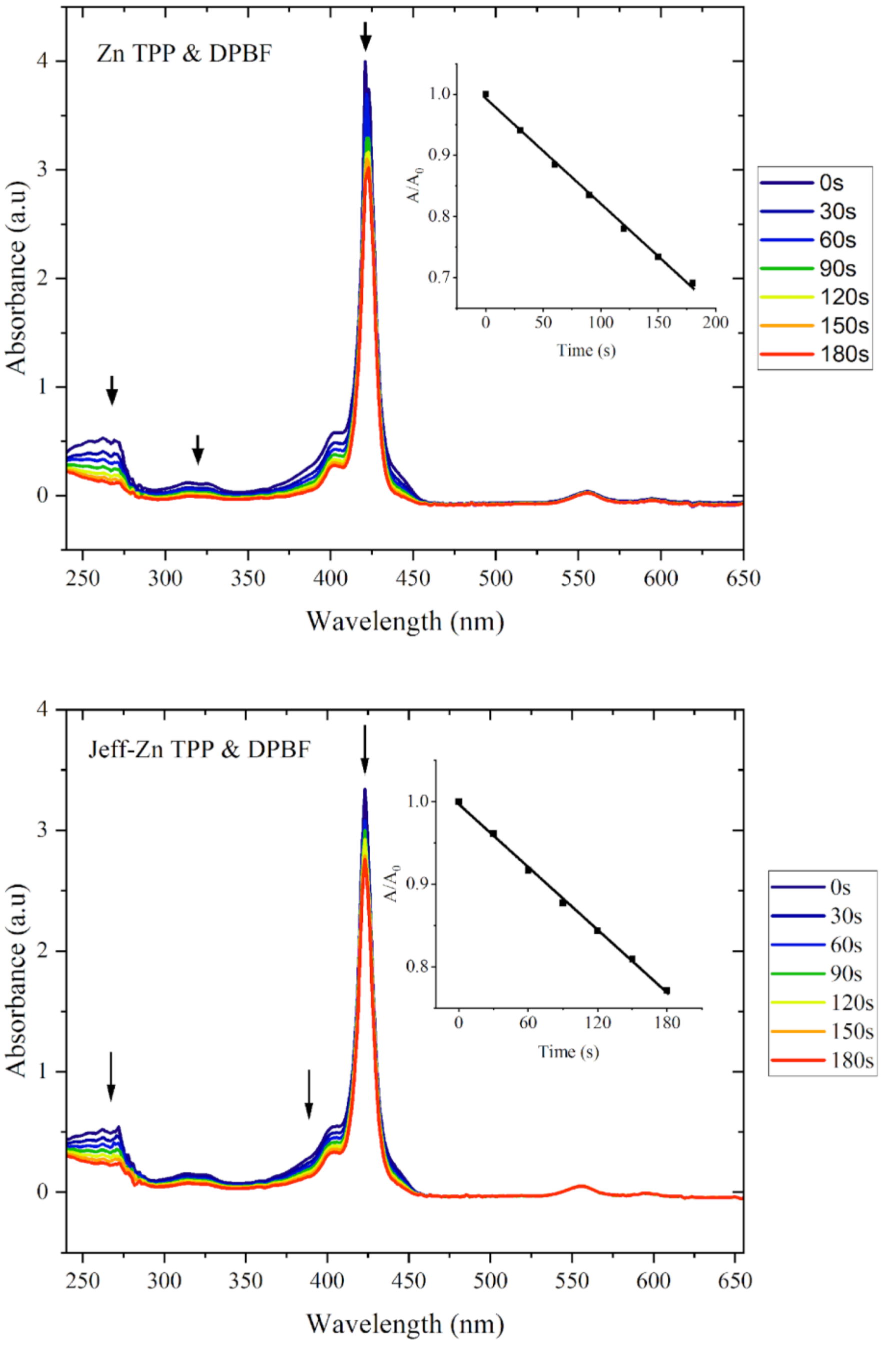

Figure S19. Time-dependent absorption spectra at 415 nm of DPBF (in DMF) in the presence of Zn-TPP, and Jeff-ZnTPP polymer. Insert: The decay rate of the absorption of DPBF at 415 nm during irradiation of Zn- Zn-TPP, and the Jeff-ZnTPP polymer.

Time resolved photophysical measurements.

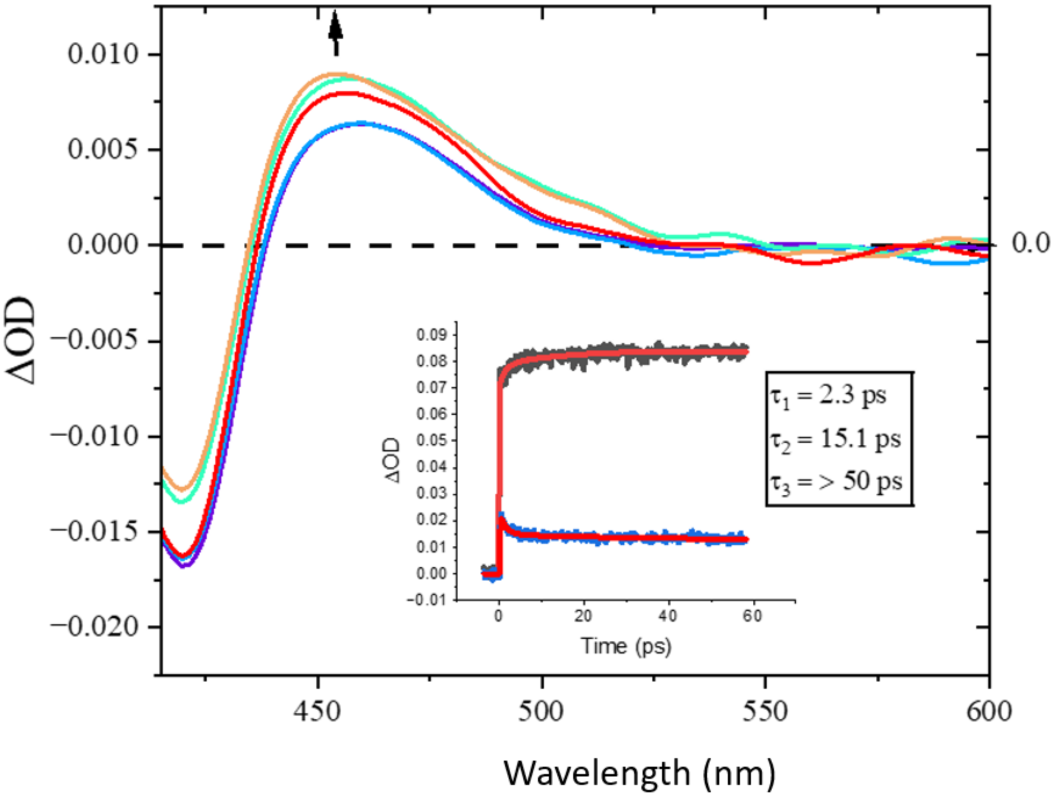

Figure S20. The spectral transient absorption profile for Jeff-ZnTPP in Ethyl acetate over a 1500 ps time range, with the insert displaying the kinetic time traces for Jeff-Zn TPP recorded at 450 nm (red datapoints) and 650 nm (blue datapoints), displayed over 50 ps.

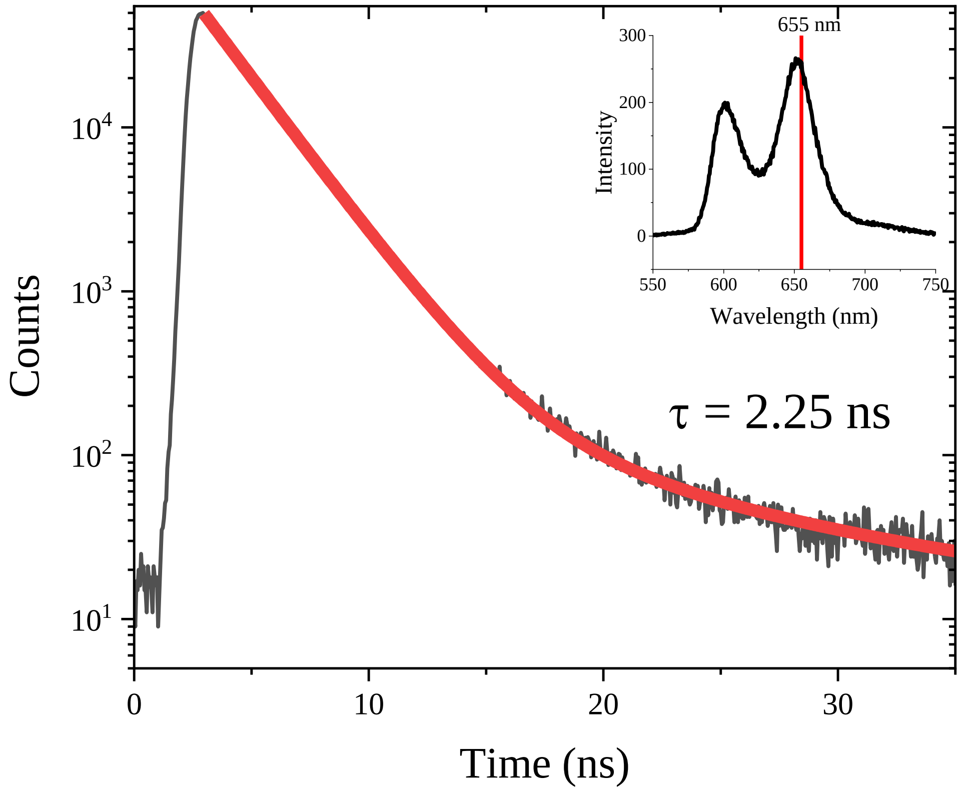

Figure S21. Fluorescence decay profile of Jeff-ZnTPP (λ_{ex} = 375 nm) in ethyl acetate over a 35 ns time range. The red solid curve represents the fitted curve with the insert displaying the emission spectrum.

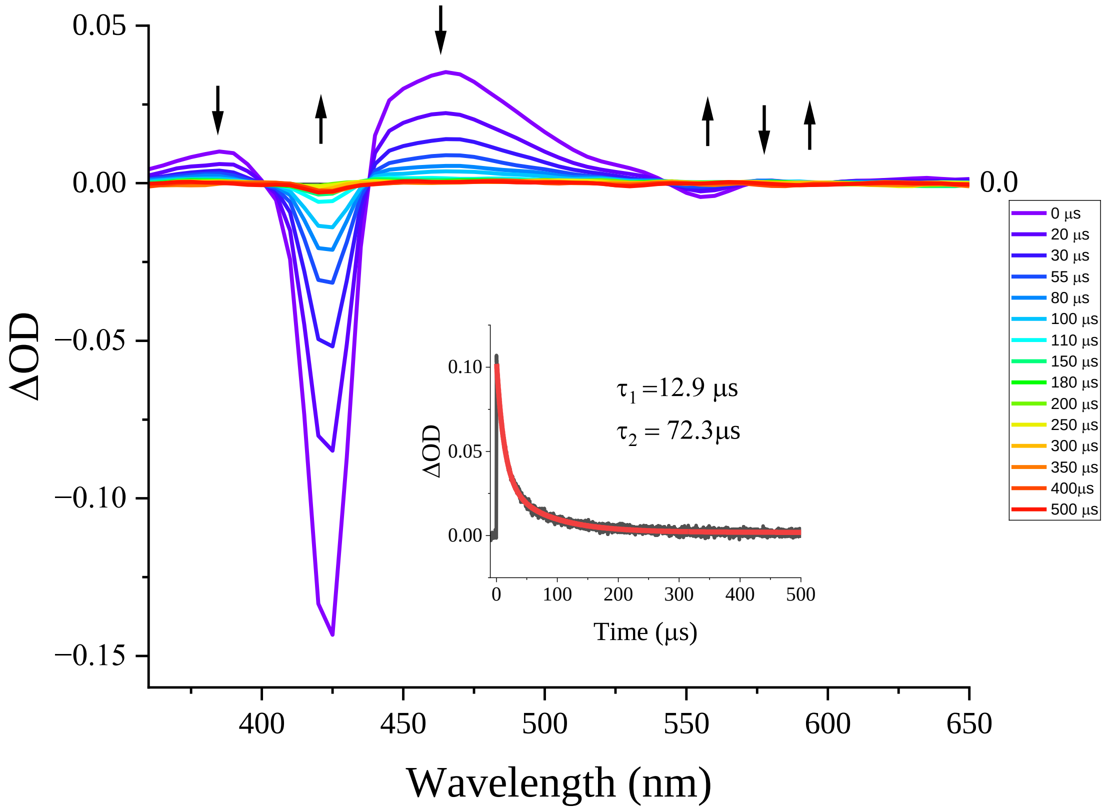

Figure S22. The spectral transient absorption profile for Jeff-ZnTPP in THF over a 500 μs time range with the insert displaying the kinetic time trace for Jeff-ZnTPP recorded at 550 nm in THF, recorded over a 500 μs time range.

DNA binding studies

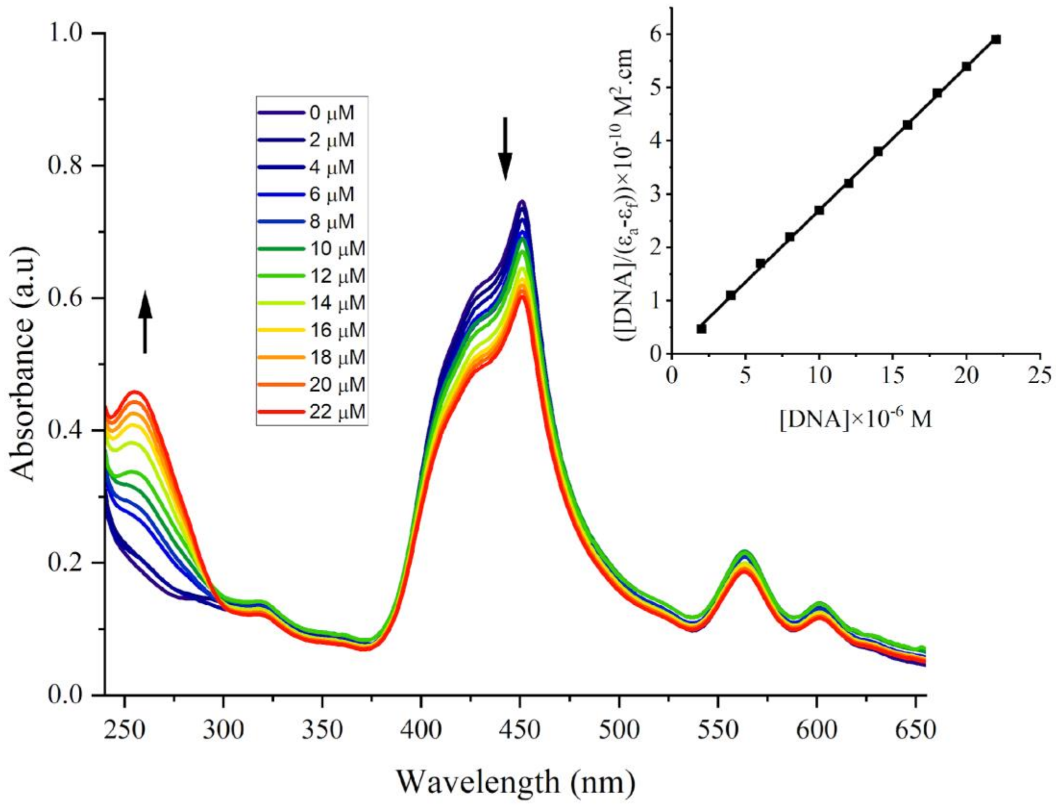

Figure S23. Absorption spectra of Zn TPP (10 μM) and in Tris-HCl buffer upon addition of CT DNA (0-24 μM). Arrow shows that the absorption intensities decrease upon increasing DNA concentration. Insert: Plot of [DNA] / (ε_a - ε_f) versus [DNA] for the titration of the Zn TPP and with CT DNA.

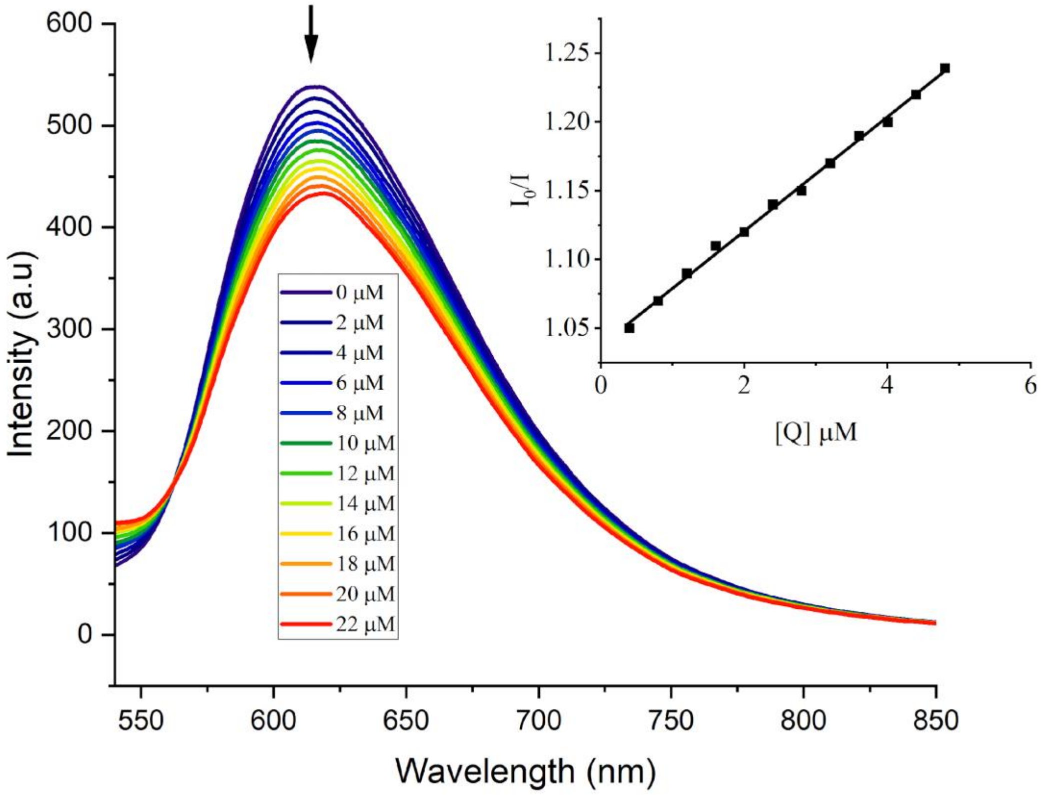

Figure S24. Fluorescence quenching curves of EB bound to DNA in the presence of Zn TPP in Tris-HCl buffer. [DNA] = 5 µM, [EB] = 5 µM and [compounds] = 0-24 µM. Insert: Stern–Volmer plot of fluorescence titrations of ZnTPP with CT DNA.

Protein binding studies

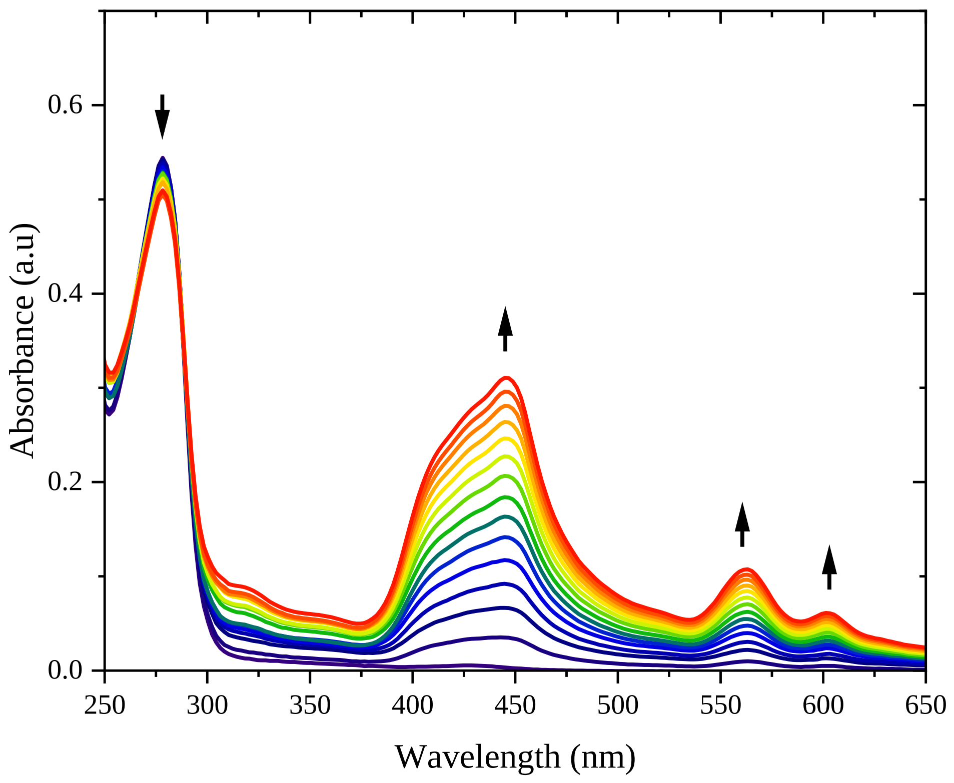

Figure S25. UV-vis absorption of BSA (10 μM) in PBS solution in the presence of different amounts (0-24 μM) of ZnTPP.

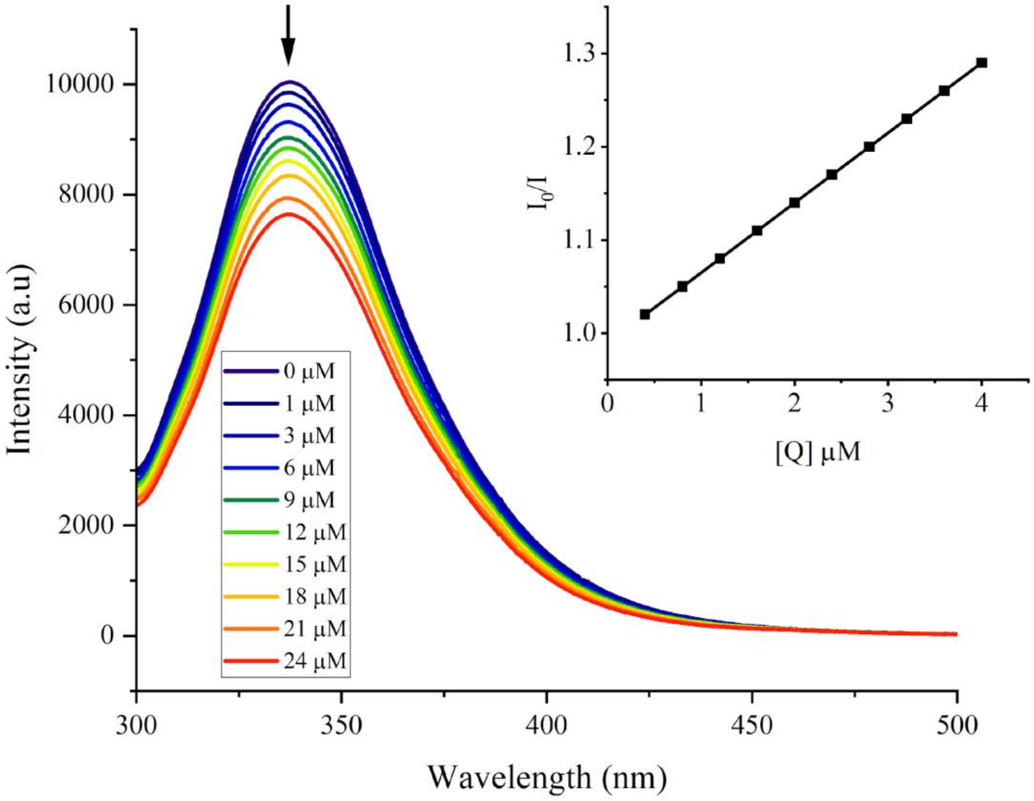

Figure S26. Fluorescence quenching curves of BSA (10 µM) in PBS solution in the presence of different amounts (0-24 µM) of ZnTPP. Insert: Stern–Volmer plot of the fluorescence titrations.

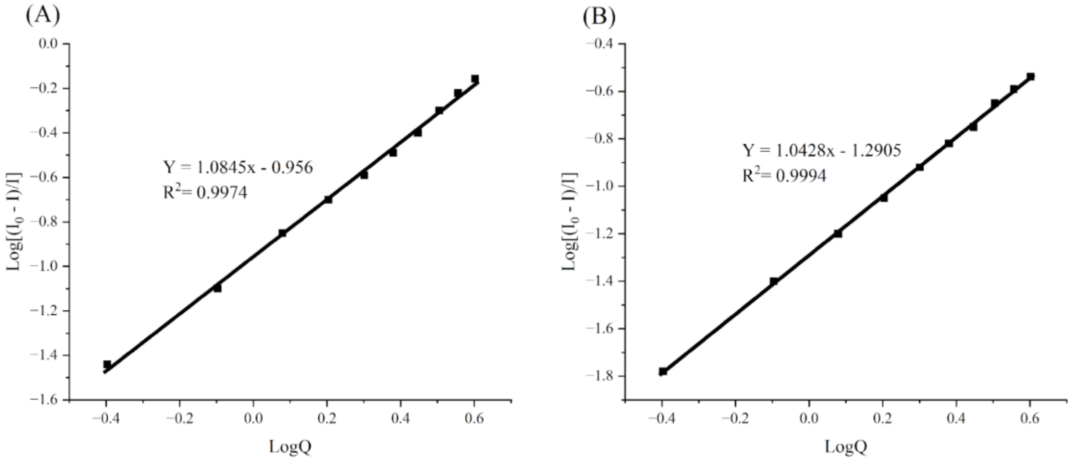

Figure S27. Scatchard plot of the fluorescence titrations of (a) Jeff-ZnTPP polymer and (b) Zn TPP (0-24 µM) with BSA (10 µM).

Antimicrobial activity investigation

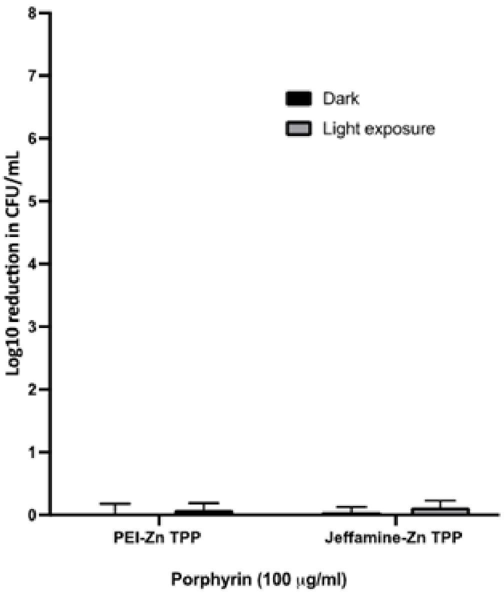

Figure S28. Antimicrobial activity of porphyrins against *E.coli*. Bacterial suspensions (~10^5 CFU/ml) were incubated with Zn-TPP-COOH, Jeffamine-Zn TPP polymer or PEI-Zn TPP polymer for 30 minutes either in dark conditions or exposed to light (λ=430 and 660 nm, 21.93 mW/cm^2). Bactericidal activity is shown in log_{10} reduction in colony forming units (CFUs)/ml. Assays were performed three times and values shown are the mean ± standard error of the mean.

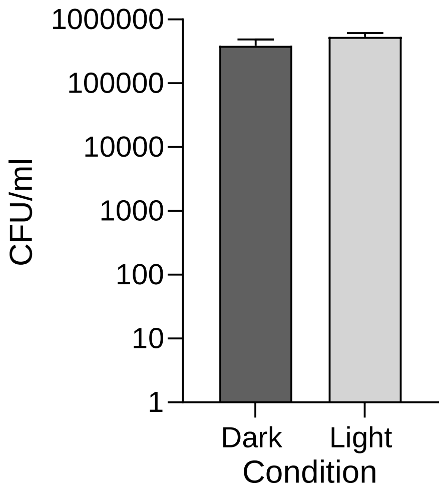

Figure S29. Comparison of the number of bacteria (*S. aureus* 25923) remaining following incubation in 10% DMSO, 90 % PBS in the dark for 60 min *Vs* or irradiated with light for 60 min. Light source (λ = 420 and 660 nm, 21.93 mW/cm^2) was located at distance of 25cm from the plate.

Table S2. Bacterial strains and isolates used in the present study. Table adapted from Doherty et al, 2024 (Reference 1)

	Species	Reference ID	Resistances	Susceptibilities
Reference strains	*A. baumannii*	ATCC19606	N/A	AMK, CAZ, CIP, CN, MEM, TOB
	E. cloacae complex (*AmpC* only)	NCTC 13405	AMC, AMP, FOX	AMK, CAZ, CIP, CN, COL, CTX, ERT, IMP, MEM, TIG, TOB, TZP,
	E. faecium (VRE)	NCTC 12204	AMP, TEC, VAN	DAP, LNZ, Q-D
	E. faecium	NCTC 7174	N/A	N/A
	K. pneumoniae	NCTC 9633	N/A	N/A
	K. pneumoniae (CPE NDM-1, ESBL)	ATCC 2146	AMK, AMP, AMC, AZM, CAZ,<br>CIP, CN, CPD, CRO, CTX, CXM<br>ERT, FOX, IMP, MEM, NF, TET<br>TIG, TOB, SXT	COL
	P. aeruginosa	ATCC 27853	N/A	N/A
	P. aeruginosa (VIM/ESBL)	NCTC 13437	AMK, AZM, CAZ, CIP, CN, IMP<br>MEM, TOB, TZP	COL
	S. aureus (MRSA)	ATCC 43300	MET, OX	
	S. aureus	ATCC 25923	N/A	N/A
	Species	**Site of infection/ Underlying disease**		
Clinical Isolates	*P. aeruginosa*	Ankle ulcer right/PVD	Not Tested	Not Tested
	A. lwoffi	Right foot ulcer/Diabetes	CIP	AMK, CN, MEM, TOB

E. cloacae complex	Ulcer foot/Diabetes/PVD	AMK, AZM, CAZ, CIP, CN, CRO, ERT, MEM, TZP	AMP, AMC
S. aureus (MRSA)	Pus Toe/Diabetes	CIP, CN, DAP, LNZ, MUP, SXT, TEC, VAN	CLI, ERY, FOX, OX, TET
E. faecium (VRE)	BKA/Diabetes/PVD	AMP, VAN	LNZ
A. baumannii	BAL/unknown	AZM, CIP, CN, MEM, TIG, TZP, SXT	AMK, FDC
S. dysgalactiae	Foot Ulcer/Diabetes/Oncology	-	ERY, PEN, TET, SXT, VAN
S. aureus	Foot Ulcer/Diabetes	-	CIP, CLI, CN, ERY, FOX, MUP, LNZ, OX, TET, SXT, VAN
K. oxytoca	Foot Ulcer/Diabetes	AMP	AMC, AMK, AZM, CAZ, CIP, CN, CRO, CXM, ERT, FOS, MEM, SXT, TZP

AMC – Amoxicillin-Clavulanate, AMP – Ampicillin, AMK – Amikacin, AZM – Aztreonam, CAZ – Ceftazidime, CIP – Ciprofloxacin, CN – Gentamicin, COL – Colistin, CRO – Ceftriaxone, CTX – Cefotaxime, CXM – Cefuroxime, DAP – Daptomycin, ERT – Ertapenem, FOX – Cefoxitin, LNZ – Linezolid, MEM – Meropenem, OX – Oxacillin(/Flucloxacillin), Q-D – Quinpristin-Dalfopristin, SXT – Trimethoprim-sulfamethoxazole, TEC – Teicoplanin, TET – Tetracyclines, TIG – Tigecycline, TOB – Tobramycin, TZP – Piperacillin-Tazobactam, VAN – Vancomycin, ERY – Erythromycin (Macrolides)

FDC – Cefiderocol, FOS – Fosfomycin, MUP- Mupirocin, BAL – Bronchoalveolar Lavage, BKA – Below Knee Amputation, PVD - Peripheral Vascular Disease, ATCC – American Type Culture Collections, NCTC – National Collection of Type Cultures

Reference: (1) Doherty, A.; Murphy, R.; Heise, A.; Fitzpatrick, F.; Fitzgerald-Hughes, D.; Antimicrobial spectrum against wound pathogens and cytotoxicity of star-arranged poly-l-lysine-based antimicrobial peptide polymers. *J. Med. Microbiol.* **2024**, *73(9)*, 001886.
